# Muscle distribution in relation to all-cause and cause-specific mortality in young and middle-aged adults

**DOI:** 10.1186/s12967-023-04008-7

**Published:** 2023-02-25

**Authors:** Chen-An Liu, Tong Liu, Yi-Zhong Ge, Meng-Meng Song, Guo-Tian Ruan, Shi-Qi Lin, Hai-Lun Xie, Jin-Yu Shi, Xin Zheng, Yue Chen, Liuyi Shen, Li Deng, Han-Ping Shi

**Affiliations:** 1 Department of Gastrointestinal Surgery, Department of Clinical Nutrition, Beijing Shijitan Hospital, Capital Medical University, Beijing, 100038 China; 2 National Clinical Research Center for Geriatric Diseases, Xuanwu Hospital, Capital Medical University, Beijing, 100053 China; 3 Key Laboratory of Cancer FSMP for State Market Regulation, Beijing, 100038 China; 4 Beijing International Science and Technology Cooperation Base for Cancer Metabolism and Nutrition, Beijing, 100038 China; 5 Shanxi Medical University, Taiyuan, Shanxi 030001 China

**Keywords:** Muscle, Distribution, Mortality, Age, Sex, NHANES

## Abstract

**Background:**

The relationship between muscle and prognosis, especially that between muscle distribution across different body parts, and the related prognosis is not well established.

**Objective:**

To investigate the relationship between muscle distribution and all-cause and cause-specific mortality and their potential modifiers.

**Design:**

Longitudinal cohort study. C-index, IDI, and NRI were used to determine the best indicator of prognosis. COX regression analysis was performed to explore the relationship between variables and outcomes. Interaction and subgroup analyses were applied to identify the potential modifiers.

**Participants:**

A total of 5052 participants (weighted: 124,841,420) extracted from the NHANES 2003–2006 of median age 45 years and constituting 50.3% men were assessed. For validation, we included 3040 patients from the INSCOC cohort in China.

**Main measures:**

Muscle mass and distribution.

**Key Results:**

COX regression analysis revealed that upper limbs (HR = 0.41, 95% CI 0.33–0.51), lower limbs (HR = 0.54, 95% CI 0.47–0.64), trunk (HR = 0.71, 95% CI, 0.59–0.85), gynoid (HR = 0.47, 95% CI 0.38–0.58), and total lean mass (HR = 0.55, 95% CI 0.45–0.66) were all associated with the better survival of participants (P _trend_ < 0.001). The changes in the lean mass ratio of the upper and lower limbs and the lean mass ratio of the android and gynoid attenuated the protective effect of lean mass. Age and sex acted as potential modifiers, and the relationship between lean mass and the prognosis was more significant in men and middle-aged participants when compared to that in other age groups. Sensitive analyses depicted that despite lean mass having a long-term impact on prognosis (15 years), it has a more substantial effect on near-term survival (5 years).

**Conclusion:**

Muscle mass and its distribution affect the prognosis with a more significant impact on the near-term than that on the long-term prognosis. Age and sex acted as vital modifiers.

**Supplementary Information:**

The online version contains supplementary material available at 10.1186/s12967-023-04008-7.

## Introduction

Owing to the ease of measurement and widespread dissemination, body mass index (BMI) has become the *de facto* standard for defining overweight, obesity, and other sub-health states [1]. However, recently, the “obesity paradox”, which is based on the BMI, has been extensively studied and more accepted against the simple BMI measure. In comparison to patients with heart failure and cancer, overweight or obese patients have better quality and longer life [2–4]. Therefore, it has become increasingly unacceptable to judge the prognosis of weight-related disorders merely based on BMI. High fat or high lean mass can increase the BMI, thereby affecting the prognosis. Fat accumulation elevates pro-inflammatory factors and induces abnormal changes in the body’s metabolic pattern [5]. In contrast, an increase in the total lean mass is inextricably linked to an improved quality of life and better prognosis [6]. However, this point is controversial as some studies report different relationships between lean body mass with all-cause mortality and cause-specific mortality [7–11]. Thus, it is very early to rely entirely on the total lean body mass to discriminate participants’ prognosis, considering that the abnormal distribution of lean body mass may have an unexpected impact on the patient’s prognosis.

The studies published on body composition, comorbidities, and prognosis to date have not been conclusive, and the optimal body composition-derived prognostic assessment tools need to be explored further. The increasing trend of obesity among young and middle-aged adults has overtaken older adults and children as one of the worst global health burdens [12, 13]. However, only a few studies have focused on the sex and age-structure-based role of muscle distribution and mortality. In this context, the present study aimed to use lean mass data of young and middle-aged adults through dual-energy X-ray absorptiometry (DEXA) (from the National Health and Nutrition Examination Survey (NHANES) 2003–2006) and bio-impedance analysis (BIA) (from the Investigation on Nutrition Status and its Clinical Outcome of Common Cancers (INSCOC) cohort) to evaluate the best lean-derived prognostic factor. Further, we explored the relationships between muscle distribution and prognosis as well as the potential modifiers that affect these relationships.

## Methods

### Study design and study population

The study population was drawn from NHANES 2003–2006, which is an ongoing research program designed to assess the health and nutritional status of US adults and children. To generate reliable population data, NHANES uses a complex, stratified, multistage sample-sampling design with the approval of the NCHS ethics review board and after obtaining written informed consent from each participant [14]. For validation, we employed the BIA data from the INSCOC cohort, which is a multi-center (40 clinical centers), continuous, large cohort study from June 2012–2021 to explore the nutritional status and body composition of Chinese cancer patients (Additional file 1: Methods 1) [15, 16]. INSCOC was registered at chictr.org.cn (registration number: ChiCTR1800020329).

We included participants (n = 5700) whose detailed lean mass data during NHANES 2003–2006 and those (n = 5667) whose detailed lean mass data during INSCOC were available. After screening 5052 participants (weighted: 124,841,420) from NHANES and 3040 patients from INSCOC aged 20–59 years were included in this study (Additional file 1: Methods 2, Fig. S1).

### Exposures

The primary variable of this study was the lean mass of different body parts, which was determined by DEXA (Hologic QDR-4500 A fanbeam densitometer, Hologic, Inc., Bedford, Massachusetts). DEXA is one of the widely accepted methods for measuring body composition. It can accurately and quickly provide the measurements of bone tissues and soft tissues [17]. In this study, we included participants’ upper limb lean mass, lower limb lean mass, trunk lean mass, total lean mass, and the lean mass of two special sites (i.e., the android and gynoid) [18] (Additional file 1: Methods 3). In addition, to avoid bias caused by height, weight, and other factors, we introduced the concept of relative lean mass, which is the lean mass/height, lean mass/weight, and lean mass/BMI.

### Covariants

The covariates of NHANES included age, sex, race, marital status, family poverty index ratio (PIR), education level, health insurance, smoking, drinking, BMI, waist circumference, hypertension, diabetes, coronary heart disease (CHD), and tumor history. We regarded participants aged 20–45 years as young and those aged 45–59 years as middle-aged. According to Chen et al., the PIR can be categorized into low- (< 1.0), middle- (1.1–3.0), and high (> 3.0) [19], and participants with BMI > 30 kg/m^2^ were categorized as obese. Therefore, we considered the interference of diet and muscle-strengthening activities (MSA) on the lean mass and prognosis and then corrected them [20, 21]. Among them, the diet quality was evaluated by the Healthy Diet Index (HEI-2015) score [22] (Additional file 1: Methods 4). As the variable information of INSCOC was slightly different from that of NHANES, its covariate information and definition have been detailed in Additional file 1: Methods 5.

### Outcomes

The follow-up data was related to the death certificate records in the national death index, and the last survey date was conducted on December 31, 2019 [23]. The follow-up data of INSCOC was June 30, 2021. In addition, to determine the relationship between muscle distribution and cause-specific mortality, we determined cardiovascular diseases (I00-I09, I11, I13, I20-I5, and I60-I69), cancer (C00-C97), and other causes of death for participants with reference to the ICD-10.

### Statistical analyses

We applied sex-specific quintiles to divide the participants. Shapiro–Wilk test was performed to determine whether the continuous variables such as age, BMI, and waist circumference of the participants obey the normal distribution; at p > 0.05, we considered that the population distribution from which the sample arises is normal. The differences in the clinical characteristics between the groups were tested by one-way analysis of variance (ANOVA; normal distribution), Kruskal–Wallis test (skewed distribution), or Chi-square test (categories data). First, we compared the differentiation of absolute lean mass and relative lean mass for predicting the prognosis and then compared them through C-index, Integrated Discrimination Improvement (IDI), and Net Reclassification Improvement (NRI). The variables with the best differentiation were applied for subsequent analyses. Kaplan–Meier curves and COX proportional regression risk model were employed to explore the relationship between muscle distribution and prognosis. In order to determine the modifiers, we conducted interactive analysis on the sex-specific quintiles of lean mass and some covariants. For modifiers, we conducted a subgroup analysis to illustrate the differences between the groups. Finally, we conducted sensitivity analyses, after excluding patients who died recently (< 18 months), and analyzed the relationship between muscle distribution and short-term and long-term mortalities. All weighted statistical analyses were conducted with the R 4.0.3 software. Two-sided P < 0.05 was considered to indicate statistical significance.

## Results

### Clinical characteristics of the study participants

A total of 5052 participants (weighted: 124,841,420) from NHANES 2003–2006 were included in the study, with a median follow-up time of 175.5 months. The median participants’ age was approximately 45 years, among which 2646 (50.3%) participants were men, 32.1% of them performed MSA, and only 6.2% received an optimal quality diet. When compared with the participants with a lower total lean mass (Q1), those with a higher total lean mass (Q5) tended to be younger, had higher BMI, had higher PIR, had higher educational levels, and accepted MSA more (Table 1). Additional file 1: Table S1 depicts the difference between the lean groups as per the different body parts. The lean mass of the limbs and gynoid gradually decreased with age, while the trunk lean mass had no significant correlation with age (P = 0.777). The android lean mass gradually increased with age (P < 0.001). A total of 3040 patients with cancer from INSCOC were enrolled, with a median follow-up time of 31.1 months. The median participants’ age was approximately 51 years, among which 1334 (43.8%) participants were men, 20.0% were cachexia, 32.3% had eating difficulties, and 47.8% were malnourished. When compared with patients with a lower total lean mass (Q1), those with a higher total lean mass (Q5) also tended to be younger and had higher BMI (Additional file 1: Table S2).

**Table 1:** Clinical characteristics of participants grouped by sex-specific of total lean mass

	Sex-specific Quintiles of total lean mass			P*
	Q1^a^	Q3^a^	Q5^#^	
N	1012	1010	1010	
Age (median, IQR)	47 [33, 55]	45 [32, 51]	42 [31, 49]	**< 0.001**
Sex (%)				
Women	482 (53.1)	481 (48.9)	481 (46.0)	**0.032**
Men	530 (46.9)	529 (51.1)	529 (54.0)	
BMI (mean (SD))	22.6 (3.0)	26.3 (3.5)	33.0 (4.4)	**< 0.001**
Waist circumference (median, IQR)	84.8 [78.0, 91.8]	94.8 [86.6, 101.4]	108.3 [101.2, 115.0]	**< 0.001**
Ethnicity (%)				
Mexican American	321 (13.1)	234 (8.6)	115 (4.8)	**< 0.001**
Other Hispanic	51 (5.9)	45 (5.3)	17 (2.2)	
Non-Hispanic White	445 (62.0)	526 (74.1)	506 (73.8)	
Non-Hispanic Black	99 (5.2)	170 (8.1)	337 (15.6)	
Other race-including multi-racial	96 (13.7)	35 (3.9)	35 (3.7)	
Marital (%)				
Married	612 (62.2)	649 (67.6)	631 (67.9)	0.285
Separated	223 (18.2)	194 (15.7)	193 (16.7)	
Never married	177 (19.6)	167 (16.7)	186 (15.4)	
Family income-poverty ratio level (%)				
0–1.0	223 (14.5)	161 (10.6)	135 (9.6)	**< 0.001**
1.1-3.0	456 (39.7)	400 (34.2)	370 (31.5)	
> 3.0	333 (45.8)	449 (55.2)	505 (58.9)	
Education (%)				
Less than high school	366 (22.5)	242 (14.5)	182 (12.1)	**< 0.001**
High school or equivalent	231 (25.4)	249 (24.2)	257 (27.4)	
College or above	415 (52.1)	519 (61.2)	571 (60.5)	
Obesity (%)				
Yes	175 (13.2)	468 (40.2)	888 (88.2)	**< 0.001**
Health insurance (%)				
Yes	759 (76.7)	744 (77.1)	809 (83.9)	**0.012**
Alcohol (%)				
Yes	701 (72.0)	717 (74.2)	735 (76.9)	0.191
Smoke (%)				
Yes	503 (50.2)	485 (49.4)	483 (48.1)	0.566
Muscle strengthening activities (%)				
Yes	236 (27.2)	315 (34.7)	335 (33.5)	**0.042**
HEI 2015(%)				
< 50	504 (52.1)	517 (52.0)	624 (62.0)	**0.003**
50–70	421 (39.6)	416 (40.9)	343 (34.1)	
> 70	87 (8.3)	77 (7.1)	43 (3.9)	
Hypertension (%)				
Yes	372 (29.2)	337 (29.1)	429 (37.7)	**0.003**
Diabetes (%)				
Yes	158 (10.6)	157 (11.3)	214 (19.1)	**< 0.001**
CHD (%)				
Yes	41 (3.1)	29 (2.1)	25 (1.9)	0.477
Cancer (%)				
Yes	88 (8.0)	59 (5.9)	56 (5.5)	0.125

The mean (SD), percentage and P values were weighted values. Results presented with bold valued were statistically significant with all p value < 0.05

^a^Women Q1, 23354.9–35406.2 g, Q3, 38627.5–42109.8 g, Q5, 46498.5–68252.9 g. Men Q1, 31887.2–50773.2 g, Q3, 55397.1–59575.6 g, Q5, 65011.2–89856.5 g

*The difference of age and waist circumference between five groups were tested by Kruskal-Wallis test. One-way Anova test was used to compare BMI. Chi-square test was used to compare other factors among five groups

### Comparison of absolute and relative lean mass in predicting prognosis

**Fig. 1:**
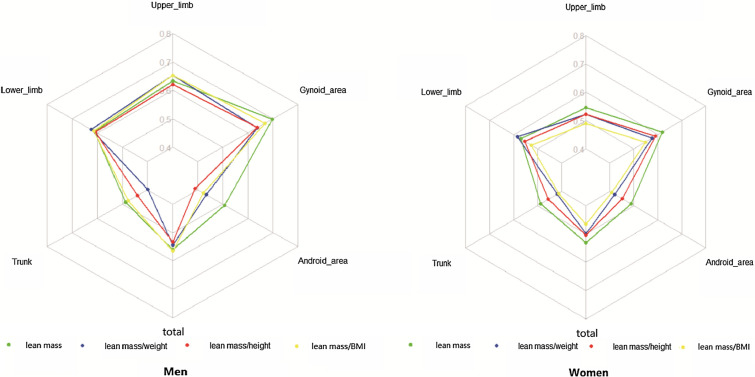
C-index of absolute and relative lean mass in predicting prognosis

As shown in Fig. 1, both men and women had the highest C-index of absolute lean mass in almost all body parts. The IDI and NRI comparisons revealed that the absolute lean mass was superior to that with other evaluation tools and it was hence used for analyses in the subsequent studies (Additional file 1: Tables S3, S4). In longitudinal comparison, the C-index of gynoid lean mass was the highest (0.72, 95% CI, 0.70–0.75).

### The relationship between lean mass distribution and all-cause mortality

The KM curve demonstrated that the sex-specific quintile of other lean mass, other than android lean mass, can differentiate between the survival of participants (Additional file 1: Fig. S2). Although KM revealed similar tendencies for both sexes, the lean mass appeared to have a more significant impact on male survival than on female survival (Q1 participants showed worse survival; Additional file 1: Fig. S2).

We further adjusted the other confounding factors (Table 2). Upper limbs (HR = 0.41, 95% CI 0.33–0.51, P < 0.001), lower limbs (HR = 0.54, 95% CI 0.47–0.64, P < 0.001), trunk (HR = 0.71, 95% CI 0.59–0.85, P < 0.001), gynoid (HR = 0.47, 95% CI 0.38–0.58, P < 0.001), and total lean mass (HR = 0.55, 95% CI 0.45–0.66, P < 0.001) were all negatively correlated with the survival of participants (Ptrend < 0.001). Although the relationship between android lean mass and survival was not apparent, the ratio between android lean mass and gynoid lean mass (A–G ratio) acted as a significant risk factor for prognosis (HR = 1.56, 95%CI 1.42–1.72, P < 0.001). Interestingly, the effects of the upper and lower limbs’ lean mass on the prognosis were different. With the increase in the lean ratio of upper and lower limbs (U-D ratio), the participants’ risk of death was lower.

**Table 2:** Hazard ratios (95% CI) of all-cause mortality according to lean mass distribution

	Events/total	Mortality rate (per 1000 person-year)	Model 1		Model 2	
			h (95%CI)	P	HR (95%CI)	P
Upper limb lean mass						
Q1	318/1012	22.49	Ref.		Ref.	
Q2	173/1010	12.26	0.62 (0.49, 0.79)	**< 0.001**	0.57 (0.43, 0.75)	**< 0.001**
Q3	128/1010	9.07	0.44 (0.33, 0.59)	**< 0.001**	0.43 (0.32, 0.58)	**< 0.001**
Q4	106/1010	7.51	0.35 (0.27, 0.47)	**< 0.001**	0.37 (0.27, 0.51)	**< 0.001**
Q5	101/1010	7.16	0.41 (0.29, 0.58)	**< 0.001**	0.32 (0.24, 0.45)	**< 0.001**
P for trend				**< 0.001**		**< 0.001**
As continuous (per SD)			0.90 (0.82, 0.98)	**0.022**	0.41 (0.33, 0.51)	**< 0.001**
Lower limb lean mass						
Q1	309/1012	21.85	Ref.		Ref.	
Q2	181/1010	12.83	0.54 (0.43, 0.68)	**< 0.001**	0.55 (0.43, 0.71)	**< 0.001**
Q3	142/1010	10.06	0.40 (0.32, 0.49)	**< 0.001**	0.45 (0.34, 0.59)	**< 0.001**
Q4	116/1010	8.22	0.38 (0.28, 0.52)	**< 0.001**	0.40 (0.29, 0.55)	**< 0.001**
Q5	78/1010	5.53	0.32 (0.23, 0.44)	**< 0.001**	0.30 (0.23, 0.40)	**< 0.001**
P for trend				**< 0.001**		**< 0.001**
As continuous (per SD)			0.84 (0.77, 0.93)	**0.001**	0.54 (0.47, 0.64)	**< 0.001**
U-L ratio						
Q1	199/1012	14.07	Ref.		Ref.	
Q2	132/1010	9.35	0.63 (0.43, 0.92)	**0.016**	0.58 (0.38, 0.89)	**0.012**
Q3	172/1010	12.19	0.91 (0.67, 1.23)	0.528	0.78 (0.54, 1.13)	0.188
Q4	159/1010	11.27	0.76 (0.58, 0.99)	**0.042**	0.57 (0.43, 0.75)	**< 0.001**
Q5	164/1010	11.62	0.74 (0.55, 0.99)	**0.041**	0.56 (0.39, 0.80)	**0.001**
P for trend				0.125		**< 0.001**
As continuous (per SD)			1.04 (0.95, 1.15)	0.367	0.75 (0.64, 0.87)	**< 0.001**
Trunk lean mass						
Q1	233/1012	16.48	Ref.		Ref.	
Q2	161/1010	11.41	0.58 (0.44, 0.76)	**< 0.001**	0.64 (0.48, 0.85)	**0.002**
Q3	158/1010	11.20	0.64 (0.48, 0.86)	**0.003**	0.65 (0.45, 0.92)	**0.017**
Q4	146/1010	10.35	0.60 (0.46, 0.79)	**< 0.001**	0.58 (0.42, 0.80)	**0.001**
Q5	128/1010	9.07	0.53 (0.39, 0.73)	**< 0.001**	0.49 (0.32, 0.75)	**0.001**
P for trend				**< 0.001**		**< 0.001**
As continuous (per SD)			0.97 (0.88, 1.07)	0.554	0.71 (0.59, 0.85)	**< 0.001**
Total lean mass						
Q1	275/1012	19.45	Ref.		Ref.	
Q2	173/1010	12.26	0.55 (0.41, 0.73)	**< 0.001**	0.56 (0.42, 0.76)	**< 0.001**
Q3	143/1010	10.13	0.49 (0.37, 0.64)	**< 0.001**	0.50 (0.37, 0.67)	**< 0.001**
Q4	136/1010	9.63	0.49 (0.38, 0.65)	**< 0.001**	0.47 (0.33, 0.67)	**< 0.001**
Q5	99/1010	7.02	0.41 (0.29, 0.59)	**< 0.001**	0.34 (0.23, 0.50)	**< 0.001**
P for trend				**< 0.001**		**< 0.001**
As continuous (per SD)			0.92 (0.83, 1.01)	0.074	0.55 (0.45, 0.66)	**< 0.001**
Android lean mass						
Q1	179/1012	12.66	Ref.		Ref.	
Q2	163/1010	11.55	0.70 (0.51, 0.96)	**0.027**	0.67 (0.46, 0.99)	**0.043**
Q3	160/1010	11.34	0.72 (0.53, 0.98)	**0.037**	0.79 (0.59, 1.04)	0.097
Q4	158/1010	11.19	0.64 (0.47, 0.88)	**0.005**	0.63 (0.42, 0.94)	**0.023**
Q5	166/1010	11.76	0.79 (0.57, 1.09)	0.147	0.77 (0.50, 1.19)	0.237
P for trend				0.194		0.222
As continuous (per SD)			1.05 (0.95, 1.17)	0.338	0.96 (0.80, 1.15)	0.674
Gynoid lean mass						
Q1	335/1012	23.69	Ref.		Ref.	
Q2	171/1010	12.12	0.47 (0.36, 0.61)	**< 0.001**	0.50 (0.39, 0.65)	**< 0.001**
Q3	139/1010	9.85	0.40 (0.33, 0.50)	**< 0.001**	0.42 (0.32, 0.54)	**< 0.001**
Q4	107/1010	7.58	0.36 (0.27, 0.48)	**< 0.001**	0.37 (0.25, 0.53)	**< 0.001**
Q5	74/1010	5.24	0.29 (0.20, 0.42)	**< 0.001**	0.26 (0.17, 0.39)	**< 0.001**
P for trend				**< 0.001**		**< 0.001**
As continuous (per SD)			0.79 (0.71, 0.88)	**< 0.001**	0.47 (0.38, 0.58)	**< 0.001**
A-G ratio						
Q1	41/1012	2.90	Ref.		Ref.	
Q2	59/1010	4.18	1.06 (0.73, 1.54)	0.760	1.02 (0.69, 1.51)	0.924
Q3	108/1010	7.65	1.24 (0.80, 1.93)	0.342	1.11 (0.67, 1.86)	0.681
Q4	204/1010	14.46	2.32 (1.50, 3.58)	**< 0.001**	1.79 (1.08, 2.96)	**0.023**
Q5	404/1010	28.63	4.81 (3.32, 6.95)	**< 0.001**	3.16 (1.98, 5.03)	**< 0.001**
P for trend				**< 0.001**		**< 0.001**
As continuous (per SD)			1.83 (1.67, 2.01)	**< 0.001**	1.56 (1.42, 1.72)	**< 0.001**

Model 1 was adjusted for age

Model 2 was adjusted for, age, sex, race/ethnicity, education level, marital status, family income-poverty ratio level, hypertension, CHD, diabetes, cancer, smoke, covered by health insurance, alcohol, BMI, waist, muscle strengthening activities, HEI 2015

All quintiles (Q1–5) were represented by sex-specific quintiles. Results presented with bold valued were statistically significant with all p value < 0.05

We resegregated the sample into male and female subsets to account for possible sex differences in the lean mass prognosis (Additional file 1: Fig. S3). The men’s lean mass was more obviously inversely related to survival and had a lower HR value (as continuous, per SD).

### The relationship between lean mass distribution and cause-specific mortality

Similar to the results for all participants, the reduction in the mortality rate in the cardiovascular disease population was related to the high appendicular lean mass (upper limbs, HR = 0.38, 95% CI 0.29–0.51, P < 0.001, lower limbs, HR = 0.60, 95% CI 0.45–0.80, P < 0.001), U–D ratio (HR = 0.60, 95% CI 0.47–0.77, P < 0.001), trunk lean mass (HR = 0.74, 95% CI 0.55–0.99, P = 0.040), gynoid lean mass (HR = 0.44, 95% CI 0.35–0.56, P < 0.001), and total lean mass (HR = 0.58, 95% CI 0.43–0.78, P < 0.001), but was opposed to a high android lean mass (HR = 1.85, 95% CI 1.56–2.20, P < 0.001) and A–G ratio (HR = 1.66, 95% CI 1.44–1.91, P < 0.001). As for tumor-specific death, although there was no significant statistical significance in the muscle distribution and prognosis of most body parts [(possibly due to the low mortality (2.92 per thousand person-years)], the HR values were similar to those of all participants (Table S5).

### Interaction analysis and subgroup analysis

**Fig. 2:**
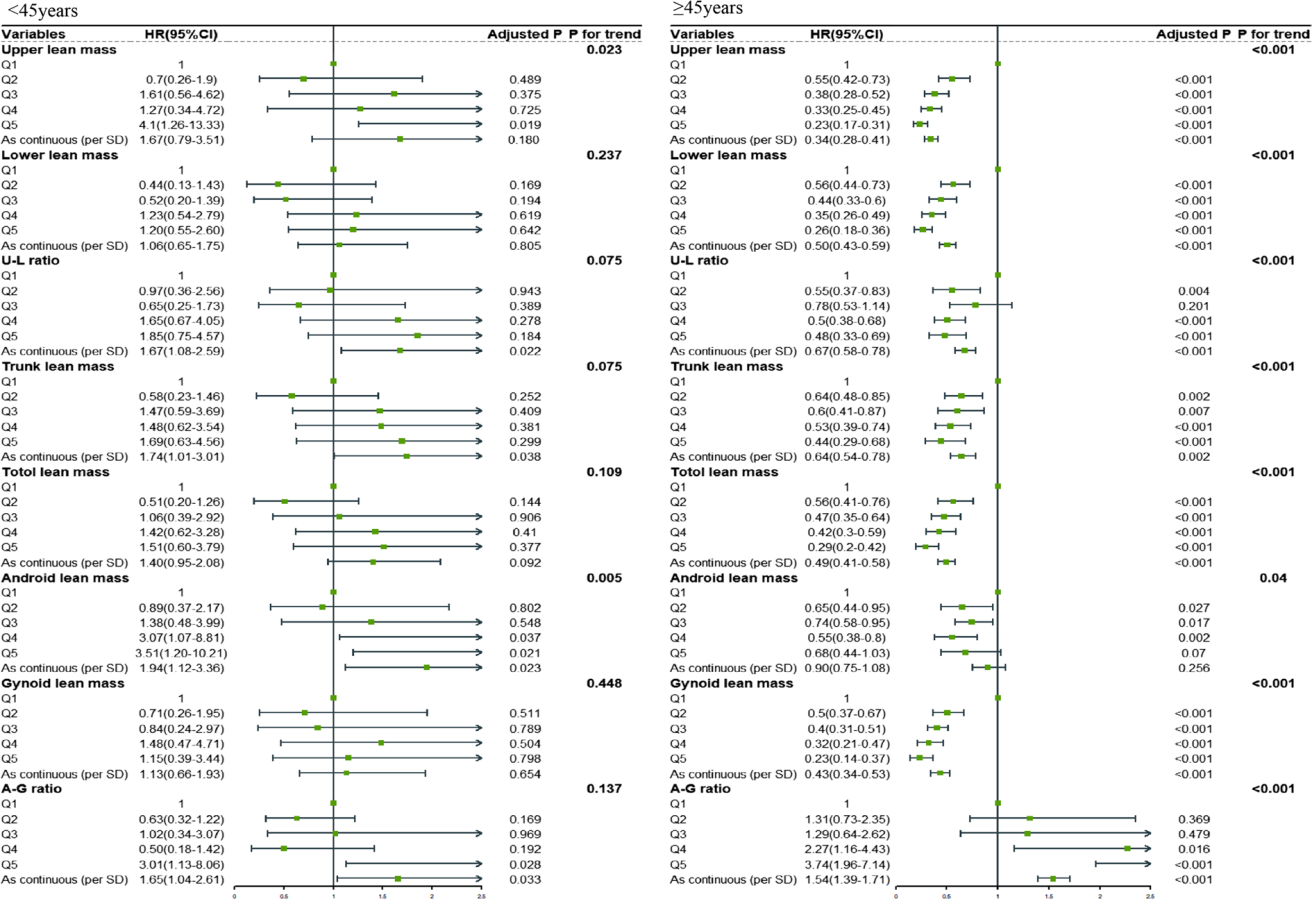
The relationship between lean mass and all-cause mortality in young and middle-aged participants

We performed an interaction and subgroup analysis of the potential modifiers to better understand how they affected our results. Table 3 indicates that the association of appendicular and total lean with all-cause mortality was affected by age (P for interaction < 0.1) and that the association of trunk and total lean mass with all-cause mortality was affected by obesity (P for interaction < 0.1). On the other hand, neither android nor gynoid lean mass interacted with any other variables. Therefore, we re-classified the participants as either middle-aged or young adult groups and as obese or non-obese groups. The correlation between lean mass and the prognosis was, unexpectedly, highly sensitive to age (Fig. 2).

**Table 3:** Interaction between the quintile of muscle mass and various factors

P for interaction	Upper limb lean mass	Lower limb lean mass	Trunk lean mass	Total lean mass	Android lean mass	Gynoid lean mass
Age	**0.001**	**0.074**	0.138	**0.090**	0.101	0.104
Race/ethnicity	0.256	0.133	0.386	0.186	0.903	0.319
Education level	0.319	0.241	0.393	0.128	0.963	0.101
Marital status	0.203	0.428	0.177	0.400	0.576	0.591
Obesity	0.299	0.127	**0.036**	**0.030**	0.411	0.225
Family income-poverty ratio level	0.973	0.640	0.682	0.850	0.778	0.736
Hypertension	0.331	0.491	0.784	0.346	0.399	0.541
CHD	0.420	0.327	0.790	0.816	0.120	0.697
Diabetes	0.720	0.409	0.613	0.579	0.347	0.172
Healthinsurance	0.142	0.506	0.642	0.590	0.877	0.381
Cancer	0.848	0.709	0.488	0.850	0.527	0.293
Alcohol	0.987	0.193	0.367	0.120	0.954	0.131
Smoke	0.754	0.248	0.603	0.919	0.314	0.681
Muscle strengthening activities	0.266	0.634	0.213	0.615	0.181	0.470
HEI2015	0.906	0.381	0.572	0.345	0.803	0.387

Results presented with bold valued were statistically significant with all P value < 0.05

There was no significant association between muscle mass at any parts and prognosis among participants aged < 45 years, whereas, for middle-aged adults, the negative linear relationship between upper limbs (HR = 0.42, 95%CI 0.34–0.53, Ptrend < 0.001), lower limbs (HR = 0.56, 95%CI 0.47–0.68, Ptrend < 0.001), U–D ratio (HR = 0.73, 95%CI 0.62–0.88, Ptrend = 0.004), trunk (HR = 0.69, 95%CI 0.54–0.89, Ptrend = 0.008), gynoid (HR = 0.46, 95%CI 0.36–0.58, Ptrend < 0.001), and total lean mass (HR = 0.54, 95%CI 0.43–0.68, Ptrend < 0.001) and prognosis appeared closer. Similarly, the middle-aged adults showed a more pronounced risk effect of the A-G ratio on the prognosis (HR = 1.53, 95%CI 1.37–1.72, Ptrend < 0.001). On the other hand, both obese and non-obese participants shared a similar relationship between lean mass and prognosis (Additional file 1: Fig. S4).

### Additional and validation analyses

All participants showed similar results in the sensitivity analyses for patients who died at the early stage of the follow-up (< 18 months) (Additional file 1: Table S6). We performed several additional analyses to gauge the influence of lean mass on near/long-term survival. Accordingly, we determined the lean body mass HR across multiple sites after 5, 10, and 15 years (Additional file 1: Table S6). Although there was no statistically significant difference among the 3 groups, the results suggested that the lean mass had a slightly greater impact on near-term survival (5 years), as was also witnessed through the HR values as continuous variables and Q5. The HR values for appendicular, the U–D ratio, trunk, gynoid, and total lean mass and prognosis were lower, while the HR value of the A–G ratio was higher. In the validation cohort, the increase in the total lean mass was significantly related to the improvement of prognosis (HR = 0.915, 95%CI 0.841–0.995, Ptrend = 0.012). We also analyzed the impact of lean mass on near/long-term survival of tumor patients. The results implied that, when compared with the 5-year survival rate, the total lean mass of patients was more closely related to that of 1-year survival (HR_1-year_=0.802, HR_5-year_=0.907).

## Discussion

Although studies have been conducted in the past to assess the correlation between the total lean mass and prognosis [12], there has been no prior work to illustrate the differences in muscle distribution and prognosis in relation to increasing age or gender. Based on the current findings, we first compared the predictive efficacy of absolute lean mass and the relative lean mass in terms of prognosis via C-index, NRI, and IDI. We also found that the absolute lean mass for any given body part was no less predictive than that of the relative lean mass. In addition, the gynoid lean mass had a stronger predictive ability relative to the total lean mass. Second, improvements in all-cause mortality and cardiovascular and cancer mortalities were predicted based on the increase in the appendicular lean mass, lean trunk mass, gynoid lean mass, and total lean mass of the participants. We also discovered that the U–D ratio acted as a protective factor against death from cardiovascular disease and all causes combined. The increase in the A–G ratio was related to the increase in risk.

Interaction and subgroup analyses revealed that differences in sex and age substantially affected the associations between lean mass and prognosis, and such relationships were more significant among men and middle-aged people. Moreover, we found that lean mass was a better predictor of near-term mortality. As with the vast majority of studies, appendicular lean, trunk lean, total lean mass, and mortality all showed linear relationships. These protective effects, however, could be nullified by an uncoordinated muscle distribution. The protective effects of lean on prognosis declined rapidly with the reduction of the U–D ratio and an increase in the A–G ratio. This abnormal phenomenon was first reported in fat distribution, and central fat accumulation was associated with an increase in all-cause mortality [24]. The significance of this distribution in lean has, however, been little discussed. Researchers have found that alterations in muscle distribution are common and meaningful among people with diabetes. During the 4.31-year follow-up, all-cause mortality was found to be positively correlated with the central distribution pattern (Increase of A-G ratio, HR = 1.35, 95%CI, 1.17–1.57), and this phenomenon is more significant in men [25]. Our findings were completely in line with this aspect. The limitation of DXEA may contribute to the problem. DXEA is unable to differentiate intramuscular fat infiltration as clearly as that by CT or MRI [26]. The infiltration of fat into muscles significantly increases local inflammation and decreases muscle functions, which significantly diminishes the protective effect of muscles on survival [27, 28]. Intriguingly, an increase in the proportion of upper and lower limb lean mass also improved survival, further emphasizing the value of upper limb lean. However, this aspect was largely ignored by research. Nonetheless, we observed that Herman et al. correlated the upper and lower limb lean mass with the prognosis. When compared with the lower limb lean mass, the upper limb lean could effectively replace the whole body muscle functions and can help predict the prognosis of elderly aged 64–95 years [29]. The U-shaped correlation may therefore be explained by the fact that some participants’ mortality did not decrease despite a significant increase in the lean mass. Possibly after accounting for abdominal obesity and the ratio of upper and lower limbs lean mass, the results can change significantly.

The loss of lean skeletal mass (sarcopenia) and strength and impaired muscle metabolism, including mitochondrial dysfunction and insulin resistance, are all common consequences of aging with far-reaching effects on the quality of life and the development of chronic diseases [30]. Mitochondria play a key role in the maintenance of muscle, but, during aging, the cellular pathways accounting for the regulation of mitochondrial metabolism (including biogenesis, dynamics, and autophagy) become vulnerable, the clearance of intracellular damaged organelles gets reduced, and the mitochondrial quality is severely impaired, because of which they cannot adapt to higher levels of oxidative stress [31]; as a result, myonuclear cell death is activated and muscle wasting ensues, leading to a concomitant systemic loss of muscle mass and function [32]. Specifically, Adam et al. reported that age-related decline in the thigh lean mass was associated with increased mortality in the elderly [33]. Interestingly, we observed the same in our investigation. There was a negative correlation between age and appendicular and gynoid lean mass, albeit there was no correlation between the trunk and android lean mass. These findings demonstrated that the lean mass in different body parts is heterogeneous. However, the heterogeneity is not only reflected in the lean mass. The interaction and subgroup analyses revealed that the correlation with prognosis was age-dependent and heterogeneous. There was almost no correlation between lean mass and prognosis in participants aged < 45 years, albeit there was a significant linear relationship among participants aged 45–64 years. Therefore, we concluded that significant changes in the lean mass and function related to age occurred between young and middle-aged people (since most studies limited the age of participants to 65 years [34]), suggesting that the association between muscle and aging is not a unique feature of the elderly. Fat infiltration into the muscles increases with age, which may explain why the quality declines after middle age [35, 36].

Considering the effects of fat infiltration, oxidative stress, and mitochondrial dysfunction on muscles and prognosis, reducing the level of oxidative stress and restoring mitochondrial functions are the best approaches to maintaining muscles and reducing the risk of death. Mild to moderate physical activity has been proven to delay age-related decline in lean mass, strength, the level of oxidative stress and inflammation, and regenerative capacity, as well as slow or prevent metabolic muscle damage [30, 37]. MSA and aerobic exercise are very effective measures [38]. Importantly, a recent review elaborated on the relationship between polyphenols and antioxidation as well as the possibility of their use as potential therapeutic drugs to alleviate neurodegeneration. Through the packaging of nanomaterials and improving the level of polyphenols in the body, the level of oxidative stress can be reduced and the mitochondrial function can be restored, which provides a new idea for a future fight against muscle aging and functional degradation [39]. In addition, due to the irreplaceable role of mitochondria in muscle metabolism, targeted mitochondrial therapy has become a new method for several diseases. Coenzyme Q10 analogs, mitochondria-related endoplasmic reticulum regulators [40], and some natural substances, such as resveratrol, can target mitochondria and effectively inhibit the MAPK and nuclear factor inflammatory pathway in the mTOR-dependent mechanism, thereby improving the muscle functions and quality safely [39, 41].

Because of the importance of accounting for sex differences in lean mass and distribution, we first split the data into quintiles based on sex. We found that women were more susceptible to lean atrophy and loss than men.

Skeletal muscle metabolism, muscle protein turnover, satellite cell content and proliferation, hormone interaction, mitochondrial function, and other related factors contribute to the disparity in the body lean percentage between sexes [42, 43]. Although previous research has linked the loss of muscle to lower bone density in women [44] and an increased risk of metabolic syndrome [45], our results indicated that changes in lean mass have a greater impact on male mortality. This finding is consistent with that of a longitudinal cohort study by Graf et al. They followed up on the prognosis of 3181 patients for > 10 years and discussed the relationship between their body composition and mortality. After adjusting for the effects of various complications, they found that lean mass had no special predictive effect on women, especially elderly women (HR = 1.05, 95% CI 0.73–1.49) [46]. Kuk et al. reported that the fat-free mass index had little predictive effect on women’s mortality [46]. When compared to studies that predicted lean mass according to a formula described elsewhere [12, 48] and those that used indirect measurement [49], the present results are more reliable and stable.

Our results may change the nutritionists’ understanding of lean mass across body regions and sexes. From the perspective of public health, we should pay attention to muscle before the age of 45 years or even earlier rather than only focusing on the elderly. Scientific MSA is reasonable, and adjusting the proportion of muscle distribution and increasing the muscle mass of specific parts may significantly improve people’s prognosis. Second, these results provide new ideas for the future basic research direction. In people of different ages, sexes, and diseases, muscle metabolism, mitochondrial function, and oxidative stress levels may differ. It is therefore necessary to describe the metabolic mode and further explore the underlying mechanism. In addition, reducing inflammation, removing oxygen-free radicals, and targeting mitochondrial therapy may provide a potential therapeutic direction for improving muscle functions and even prognosis in the future.

The following are some of the strengths of this study. First, these findings were obtained from a large sample of long-term follow-up cohorts and fully adjusted for a range of confounding factors, such as HEI-2015 and MSA. Second, we focused on the underappreciated issues of muscle distribution problems, such as prognostic value and population differences. Third, we described the muscle distribution and near- and long-term survivals. Although lean mass may have a more than 15-year impact on the prognosis, it showed a more significant impact on survival in the last 5 years.

This study has the following limitations. First, it is unclear as to which type of activity was performed and for how long before and after MSA. Second, dietary data were summarized from the participants’ memories, which may be subject to recall bias. Third, it is unclear whether the participants used drugs that may affect muscle functions, such as antioxidants. Finally, we assessed the participants’ long-term outcomes to collect data on their survival. In nearly 15 years of the median follow-up duration, the clinical characteristics of an individual may change significantly. The association between a lean mass distribution and long-term survival may thus be better described if we can obtain the trajectory of some indicators.

## Conclusion

In summary, absolute lean mass is one of the best predictors of prognosis. We noted a correlation between appendicular, trunk, gynoid, and total lean mass and the prognosis. As a prognostic indicator, other characteristics did not affect the gynoid lean mass. Appendicular, trunk, and total lean mass have different associations with prognosis depending on sex and age, and these differences emerge earlier in life, around 45 years rather than 65 years. The protective effects of lean mass are offset by an imbalance in the U–D and A–G ratios. Finally, the lean mass affected the short-term survival (5 years) more than the long-term prognosis (15 years). It is necessary to study the mechanism of muscle differentiation in different types of people. Mitochondrial targeted therapy may become a potential treatment method to resist muscle aging in the future.

## Supplementary Information

**Additional file 1: TableS1.** Clinical characteristics of participants grouped by sex-specific ofdifferent regional lean mass. **Table S2.** Clinical characteristics of participants grouped bysex-specific of total lean mass in the INSCOC cohort. **Table S3.**Comparison of discrimination of all-cause mortality with different lean mass inmen. **Table S4.** Comparison of discrimination of all-cause mortality withdifferent lean mass in women. **Table S5.** Hazards ratio (95% CI) forcause-specific mortality of lean mass. **Table S6.** Additional analyses. **Table S7.** The relationshipbetween total lean mass and prognosis of patients with cancer in the INSCOCcohort. **FigureS1.** Flow chart of research design (NHANES and INSCOCcohort). **Figure S2.** Kaplan-Meier Curves of sex-specific quintiles of leanmass. **Figure S3.** The relationship betweenlean mass and all-cause mortality in different sexes. Figure S4 Therelationship between lean mass and all-cause mortality in young and middle-agedparticipants. 

## Data Availability

The datasets used during the current study are available from the corresponding author on reasonable request.
