# Supplementary material for: Muscle distribution in relation to all-cause and cause-specific mortality in young and middle-aged adults
Source: J Transl Med. 2023 Feb 25;21:154. doi: 10.1186/s12967-023-04008-7 (PMC9960213; doi:10.1186/s12967-023-04008-7)

Additional file 1: Methods

1. See protocol of INSCOC for details: https://journals.lww.com/pn/Fulltext/2022/11000/Extension_protocol_for_the_Investigation_on.7.aspx

2.Excluded those who (1) lacked data on family poverty index ratio (PIR), marital status, education level, and health insurance; (2) lacked data on BMI and waist circumference; (3) lacked data on smoking habits, alcohol consumption, and comorbidities; (4) lacked data on muscle strengthening activities and diet. In addition, we excluded individuals who lacked follow-up and survival data.

3.Android lean mass is the area around the Waist circumference between the mid-point of the lumbar spine and the top of the pelvis, while the gynoid area lies roughly between the head of the femur and mid-thigh [1].

**[1]** Shepherd JA, Fan B, Lu Y et al. A multinational study to develop universal standardization of whole-body bone density and composition using GE Healthcare Lunar and Hologic DXA systems. J Bone Miner Res 2012; 27: 2208-2216.

4.The diet quality was evaluated by the Healthy Diet Index (HEI-2015) score, with a total score of 0–100, and the score was positively correlated with the diet quality. We calculated the HEI-2015 based on the average total nutrient intake in 2 days, and, according to the results of Wang et al., <50, 50–70, and >70 were classified as inadequate, average, and optimal, respectively [2].

**[2]** Wang K, Zhao Y, Nie J et al. Higher HEI-2015 Score Is Associated with Reduced Risk of Depression: Result from NHANES 2005-2016. Nutrients 2021; 13.

5.In the INSCOC cohort, we used Bio-impedance analysis(BIA) to measure the body composition of patients. Due to the limitations of the BIA, we can only obtain the total muscle mass information of patients. With this information, we included 3040 patients in the INSCOC cohort who have received BIA measurements as the validation cohort to supplement and verify some results of this manuscript. It should be mentioned that in the analysis process, in addition to most of the factors included in the NHANES database (sex, age, ethnicity, marital, education, alcohol, hypertension, CHD, family history of tumor), we also used residence, tumor stage, tumor type, surgery, chemotherapy, radiotherapy, urea nitrogen, albumin, hand grip strength, mid-arm muscle circumference, calf circumference, eating difficulties, physical activity, Karnofsky performance status, cachexia, and malnutrition to correct the prognosis model to avoid the impact of confounding factors. Cachexia was definined and classified based on an international consensus with the following criteria: (1) a weight loss >5% over past 6 months (in absence of simple starvation); or (2) BMI < 20 and any degree of weight loss >2%; or (3) appendicular skeletal muscle index consistent with sarcopenia and any degree of weight loss >2%. The assessment of skeletal muscle depletion was used by mid-upper arm muscle area by anthropometry (men <32 cm^2^, women <18 cm^2^) [3]. The diagnosis of malnutrition was based on Patient-Generated Subjective Global Assessment score, a score greater than or equal to 4 was considered as malnutrition [4].

**[3]** Fearon K, Strasser F, Anker SD, et al. Definition and classification of cancer cachexia: an international consensus. Lancet Oncol. 2011;12(5):489-95.

**[4]** Fu Z, Zhang R, Wang KH, et al; INSCOC Study Group. Development and validation of a Modified Patient-Generated Subjective Global Assessment as a nutritional assessment tool in cancer patients. J Cachexia Sarcopenia Muscle. 2022;13(1):343-354.

Table S1 Clinical characteristics of participants grouped by sex-specific of different regional lean mass

|  |  | Quintiles of upper limb lean mass | | |  |
| --- | --- | --- | --- | --- | --- |
|  | level | Q1 | Q3 | Q5 | P* |
| N |  | 1013 | 1009 | 1010 |  |
| Age (median, IQR) |  | 48.00 [35.00, 55.00] | 45.00 [32.00, 51.00] | 41.00 [32.00, 49.00] | **<0.001** |
| Sex (%) | Women | 482(56.2) | 481(49.8) | 481(45.2) | **0.001** |
|  | Men | 531(43.8) | 528(50.2) | 529(54.8) |  |
| BMI (mean (SD)) |  | 23.23(3.34) | 26.58(4.16) | 32.31(4.65) | **<0.001** |
| Waist circumference  (median, IQR) |  | 86.50 [79.43, 94.40] | 94.30 [85.88, 102.20] | 105.55 [97.50, 113.00] | **<0.001** |
| Ethnicity (%) | Mexican American | 271(10.3) | 230( 8.7) | 144( 6.2) | **<0.001** |
|  | Other Hispanic | 51( 5.5) | 42( 4.4) | 26( 3.5) |  |
|  | Non-Hispanic White | 538(69.6) | 540(75.9) | 404(66.3) |  |
|  | Non-Hispanic Black | 69( 3.0) | 168( 7.9) | 404(20.5) |  |
|  | Other Race - Including Multi-Racial | 84(11.5) | 29( 3.1) | 32( 3.6) |  |
| Marital (%) | Married | 607(63.4) | 659(69.5) | 629(68.6) | 0.413 |
|  | Separated | 231(18.1) | 192(15.1) | 186(16.3) |  |
|  | Never married | 175(18.5) | 158(15.4) | 195(15.1) |  |
| Family income-poverty ratio level (%) | 0-1.0 | 199(12.6) | 180(12.2) | 155(11.0) | 0.484 |
|  | 1.1-3.0 | 440(36.2) | 388(32.8) | 388(33.7) |  |
|  | >3.0 | 374(51.3) | 441(55.0) | 467(55.3) |  |
| Education (%) | Less than high school | 322(19.2) | 241(14.7) | 215(14.0) | **0.012** |
|  | High school or equivalent | 228(23.9) | 255(26.7) | 269(29.2) |  |
|  | College or above | 463(56.9) | 513(58.6) | 526(56.8) |  |
| Obesity (%) | yes | 231(20.1) | 475(42.6) | 808(81.8) | **<0.001** |
| Health insurance (%) | yes | 794(80.7) | 760(79.8) | 787(82.6) | 0.070 |
| Alcohol (%) | yes | 715(72.3) | 733(74.3) | 718(76.6) | 0.369 |
| Smoke (%) | yes | 510(48.4) | 495(50.7) | 461(47.0) | 0.412 |
| Muscle strengthening activities (%) | yes | 239(29.4) | 297(33.1) | 349(34.3) | 0.415 |
| HEI 2015(%) | <50 | 481(49.5) | 538(53.2) | 624(63.3) | **<0.001** |
|  | 50-70 | 430(40.8) | 396(39.5) | 343(33.0) |  |
|  | >70 | 102( 9.7) | 75( 7.2) | 43( 3.7) |  |
| Hypertension (%) | yes | 389(31.3) | 360(30.4) | 429(37.3) | **0.012** |
| Diabetes (%) | yes | 171(11.6) | 148(11.6) | 206(18.5) | **<0.001** |
|  |  |  |  |  |  |
| CHD (%) | yes | 50( 3.8) | 35( 2.8) | 25( 1.9) | **0.017** |
| Cancer (%) | yes | 107( 8.8) | 62( 6.8) | 48( 4.9) | 0.122 |

|  |  | Quintiles of lower limb lean mass | | |  |
| --- | --- | --- | --- | --- | --- |
|  | level | Q1 | Q3 | Q5 | P* |
| N |  | 1012 | 1010 | 1010 |  |
| Age (median, IQR) |  | 49.00 [37.00, 55.00] | 45.00 [33.00, 52.00] | 40.00 [30.00, 47.00] | **<0.001** |
| Sex (%) | Women | 482(53.1) | 481(50.0) | 481(47.3) | 0.221 |
|  | Men | 530(46.9) | 529(50.0) | 529(52.7) |  |
| BMI (mean (SD)) |  | 23.05(3.33) | 26.48(3.99) | 32.37(4.86) | **<0.001** |
| Waist circumference  (median, IQR) |  | 87.30 [79.85, 95.00] | 95.00 [86.00, 102.50] | 105.90 [97.40, 113.00] | **<0.001** |
| Ethnicity (%) | Mexican American | 382(15.4) | 213( 7.8) | 89( 4.0) | **<0.001** |
|  | Other Hispanic | 47( 5.7) | 45( 4.7) | 22( 2.6) |  |
|  | Non-Hispanic White | 438(63.5) | 571(76.8) | 449(69.2) |  |
|  | Non-Hispanic Black | 68( 3.6) | 150( 6.6) | 415(20.3) |  |
|  | Other Race - Including Multi-Racial | 77(11.8) | 31( 4.0) | 35( 3.9) |  |
| Marital (%) | Married | 641(63.5) | 670(69.6) | 604(66.5) | 0.107 |
|  | Separated | 238(20.6) | 186(15.9) | 178(15.4) |  |
|  | Never married | 133(15.9) | 154(14.5) | 228(18.2) |  |
| Family income-poverty ratio level (%) | 0-1.0 | 235(15.2) | 161(10.6) | 135( 9.5) | **<0.001** |
|  | 1.1-3.0 | 478(42.4) | 408(33.6) | 369(32.1) |  |
|  | >3.0 | 299(42.3) | 441(55.8) | 506(58.4) |  |
| Education (%) | Less than high school | 403(24.5) | 246(14.5) | 157(10.4) | **<0.001** |
|  | High school or equivalent | 241(27.2) | 249(24.6) | 237(24.8) |  |
|  | College or above | 368(48.2) | 515(60.9) | 616(64.7) |  |
| Obesity (%) | yes | 256(20.0) | 475(42.0) | 813(81.7) | **<0.001** |
| Health insurance (%) | yes | 755(75.7) | 759(78.8) | 821(84.6) | **0.001** |
| Alcohol (%) | yes | 696(72.6) | 727(74.4) | 734(76.3) | **0.050** |
| Smoke (%) | yes | 534(55.1) | 501(49.7) | 429(42.8) | **<0.001** |
| Muscle strengthening activities (%) | yes | 183(22.3) | 300(32.8) | 366(36.4) | **<0.001** |
| HEI 2015(%) | <50 | 512(54.9) | 531(53.2) | 618(60.2) | 0.068 |
|  | 50-70 | 421(37.8) | 404(40.4) | 348(35.7) |  |
|  | >70 | 79( 7.3) | 75( 6.3) | 44( 4.2) |  |
| Hypertension (%) | yes | 412(32.3) | 333(29.3) | 376(32.4) | 0.276 |
| Diabetes (%) | yes | 208(14.7) | 145(11.2) | 177(15.9) | **0.016** |
| CHD (%) | yes | 46( 3.8) | 33( 2.2) | 22( 1.6) | **0.033** |
| Cancer (%) | yes | 87( 8.2) | 67( 6.5) | 53( 5.5) | 0.358 |

|  |  | Quintiles of trunk lean mass | | |  |
| --- | --- | --- | --- | --- | --- |
|  | level | Q1 | Q3 | Q5 | P* |
| N |  | 1012 | 1010 | 1010 |  |
| Age (median, IQR) |  | 45.00 [29.00, 54.00] | 45.00 [32.00, 51.00] | 44.00 [34.00, 50.00] | 0.777 |
| Sex (%) | Women | 482(51.8) | 481(49.0) | 481(46.4) | 0.068 |
|  | Men | 530(48.2) | 529(51.0) | 529(53.6) |  |
| BMI (mean (SD)) |  | 22.51(3.00) | 26.32(3.57) | 32.85(4.35) | **<0.001** |
| Waist circumference  (median, IQR) |  | 83.60 [77.55, 89.85] | 94.80 [87.50, 101.20] | 109.20 [102.60, 115.70] | **<0.001** |
| Ethnicity (%) | Mexican American | 282(11.8) | 246( 8.9) | 144( 5.3) | **<0.001** |
|  | Other Hispanic | 52( 6.0) | 36( 4.2) | 17( 2.0) |  |
|  | Non-Hispanic White | 410(58.6) | 529(75.4) | 586(79.2) |  |
|  | Non-Hispanic Black | 175( 9.8) | 171( 8.2) | 231(10.2) |  |
|  | Other Race - Including Multi-Racial | 93(13.8) | 28( 3.3) | 32( 3.3) |  |
| Marital (%) | Married | 594(60.9) | 651(68.5) | 654(69.5) | **0.003** |
|  | Separated | 199(16.5) | 191(15.8) | 198(17.0) |  |
|  | Never married | 219(22.6) | 168(15.8) | 158(13.4) |  |
| Family income-poverty ratio level (%) | 0-1.0 | 214(14.2) | 168(10.6) | 140( 9.8) | **0.001** |
|  | 1.1-3.0 | 461(40.1) | 397(33.5) | 372(30.9) |  |
|  | >3.0 | 337(45.7) | 445(55.9) | 498(59.3) |  |
| Education (%) | Less than high school | 342(21.9) | 262(16.1) | 195(12.3) | **<0.001** |
|  | High school or equivalent | 234(25.2) | 220(21.6) | 261(27.8) |  |
|  | College or above | 436(52.9) | 528(62.3) | 554(59.9) |  |
| Obesity (%) | yes | 142(10.9) | 480(41.1) | 907(88.9) | **<0.001** |
| Health insurance (%) | yes | 747(76.2) | 754(77.5) | 807(83.4) | **0.007** |
| Alcohol (%) | yes | 698(71.9) | 730(74.9) | 741(76.6) | 0.144 |
| Smoke (%) | yes | 477(48.0) | 506(50.8) | 524(51.5) | 0.217 |
| Muscle strengthening activities (%) | yes | 266(29.0) | 319(34.2) | 307(31.9) | 0.103 |
| HEI 2015(%) | <50 | 502(51.6) | 523(52.7) | 611(61.3) | **0.001** |
|  | 50-70 | 419(39.8) | 402(39.2) | 357(35.2) |  |
|  | >70 | 91( 8.6) | 85( 8.1) | 42( 3.5) |  |
| Hypertension (%) | yes | 348(27.4) | 349(28.6) | 456(40.1) | **<0.001** |
| Diabetes (%) | yes | 130( 9.4) | 164(11.7) | 246(21.2) | **<0.001** |
| CHD (%) | yes | 32( 2.4) | 37( 2.8) | 31( 2.5) | 0.928 |
| Cancer (%) | yes | 71( 6.0) | 68( 7.3) | 73( 7.3) | 0.696 |

|  |  | Quintiles of Android lean mass | | |  |
| --- | --- | --- | --- | --- | --- |
|  | level | Q1 | Q3 | Q5 | P* |
| N |  | 1012 | 1010 | 1010 |  |
| Age (median, IQR) |  | 38.00 [26.00, 51.00] | 45.00 [33.00, 51.00] | 46.00 [38.00, 51.00] | **<0.001** |
| Sex (%) | Women | 482(53.2) | 481(50.7) | 481(45.3) | **0.017** |
|  | Men | 530(46.8) | 529(49.3) | 529(54.7) |  |
| BMI (mean (SD)) |  | 22.31(2.89) | 26.48(3.25) | 33.12(4.28) | **<0.001** |
| Waist circumference  (median, IQR) |  | 81.70 [75.90, 88.55] | 94.45 [88.23, 100.10] | 110.00 [104.00, 116.20] | **<0.001** |
| Ethnicity (%) | Mexican American | 247(10.4) | 228( 8.2) | 151( 5.0) | **<0.001** |
|  | Other Hispanic | 46( 5.3) | 39( 4.7) | 19( 2.2) |  |
|  | Non-Hispanic White | 437(62.5) | 528(74.5) | 582(79.3) |  |
|  | Non-Hispanic Black | 197(10.6) | 185( 8.9) | 224( 9.9) |  |
|  | Other Race - Including Multi-Racial | 85(11.2) | 30( 3.7) | 34( 3.6) |  |
| Marital (%) | Married | 566(58.1) | 679(70.2) | 660(70.5) | **<0.001** |
|  | Separated | 169(13.3) | 180(15.5) | 219(18.4) |  |
|  | Never married | 277(28.6) | 151(14.3) | 131(11.0) |  |
| Family income-poverty ratio level (%) | 0-1.0 | 224(14.9) | 148( 9.7) | 152(10.1) | **0.002** |
|  | 1.1-3.0 | 419(37.6) | 411(33.7) | 370(30.5) |  |
|  | >3.0 | 369(47.6) | 451(56.6) | 488(59.4) |  |
| Education (%) | Less than high school | 319(20.4) | 257(14.5) | 207(13.7) | **0.014** |
|  | High school or equivalent | 210(21.8) | 223(22.9) | 261(28.2) |  |
|  | College or above | 483(57.8) | 530(62.6) | 542(58.1) |  |
| Obesity (%) | yes | 94( 6.1) | 476(41.4) | 946(93.5) | **<0.001** |
| Health insurance (%) | yes | 729(75.4) | 765(79.4) | 820(84.3) | **0.003** |
| Alcohol (%) | yes | 706(72.8) | 740(76.1) | 719(74.8) | 0.282 |
| Smoke (%) | yes | 448(45.7) | 487(48.1) | 557(54.6) | **0.013** |
| Muscle strengthening activities (%) | yes | 325(37.2) | 309(31.8) | 269(27.7) | **0.010** |
| HEI 2015(%) | <50 | 545(56.2) | 511(51.0) | 602(60.5) | **0.001** |
|  | 50-70 | 391(36.2) | 438(44.1) | 361(35.5) |  |
|  | >70 | 76( 7.6) | 61( 4.9) | 47( 4.0) |  |
| Hypertension (%) | yes | 270(21.8) | 336(28.8) | 496(44.5) | **<0.001** |
| Diabetes (%) | yes | 85( 6.0) | 150( 9.9) | 278(23.9) | **<0.001** |
| CHD (%) | yes | 34( 2.3) | 32( 2.2) | 35( 3.0) | 0.330 |
| Cancer (%) | yes | 63( 5.1) | 57( 6.3) | 76( 7.8) | 0.116 |

|  |  | Quintiles of Gynoid lean mass | | |  |
| --- | --- | --- | --- | --- | --- |
|  | level | Q1 | Q3 | Q5 | P* |
| N |  | 1012 | 1010 | 1010 |  |
| Age (median, IQR) |  | 49.00 [37.00, 57.00] | 45.00 [32.00, 51.00] | 40.00 [30.00, 47.00] | **<0.001** |
| Sex (%) | Women | 482(51.3) | 481(50.4) | 481(47.2) | 0.146 |
|  | Men | 530(48.7) | 529(49.6) | 529(52.8) |  |
| BMI (mean (SD)) |  | 22.74(3.19) | 26.38(3.66) | 32.54(4.66) | **<0.001** |
| Waist circumference  (median, IQR) |  | 86.50 [79.00, 94.20] | 94.20 [86.50, 102.00] | 106.70 [98.60, 113.90] | **<0.001** |
| Ethnicity (%) | Mexican American | 321(13.0) | 220( 8.4) | 118( 4.7) | **<0.001** |
|  | Other Hispanic | 53( 6.2) | 39( 4.5) | 20( 2.4) |  |
|  | Non-Hispanic White | 436(60.9) | 537(75.0) | 522(75.0) |  |
|  | Non-Hispanic Black | 117( 6.6) | 179( 8.1) | 316(14.4) |  |
|  | Other Race - Including Multi-Racial | 85(13.3) | 35( 4.1) | 34( 3.6) |  |
| Marital (%) | Married | 613(61.9) | 666(70.0) | 619(67.0) | **0.035** |
|  | Separated | 252(20.7) | 186(15.7) | 176(15.1) |  |
|  | Never married | 147(17.4) | 158(14.3) | 215(17.9) |  |
| Family income-poverty  ratio level (%) | 0-1.0 | 228(15.8) | 173(11.4) | 135( 9.0) | **<0.001** |
|  | 1.1-3.0 | 484(42.3) | 399(34.6) | 361(31.0) |  |
|  | >3.0 | 300(41.8) | 438(54.1) | 514(60.0) |  |
| Education (%) | Less than high school | 395(24.5) | 240(14.8) | 169(11.2) | **<0.001** |
|  | High school or equivalent | 226(25.2) | 234(23.3) | 248(26.0) |  |
|  | College or above | 391(50.2) | 536(61.9) | 593(62.8) |  |
| Obesity (%) | yes | 234(16.2) | 472(42.2) | 843(84.0) | **<0.001** |
| Health insurance (%) | yes | 777(77.8) | 759(79.3) | 802(83.6) | 0.064 |
| Alcohol (%) | yes | 683(70.4) | 726(73.2) | 745(77.6) | **0.012** |
| Smoke (%) | yes | 530(55.3) | 501(50.3) | 460(46.1) | **0.034** |
| Muscle strengthening activities (%) | yes | 215(25.6) | 303(31.6) | 362(36.5) | **0.003** |
| HEI 2015(%) | <50 | 485(52.3) | 523(51.1) | 624(62.0) | **0.001** |
|  | 50-70 | 434(38.6) | 408(41.2) | 342(34.0) |  |
|  | >70 | 93( 9.1) | 79( 7.7) | 44( 4.0) |  |
| Hypertension (%) | yes | 432(34.0) | 352(31.0) | 375(32.7) | 0.345 |
| Diabetes (%) | yes | 196(13.8) | 156(12.0) | 184(16.3) | **0.014** |
| CHD (%) | yes | 51( 4.2) | 31( 2.2) | 19( 1.4) | **0.009** |
| Cancer (%) | yes | 99( 8.4) | 66( 6.6) | 51( 5.4) | 0.223 |

*The difference of age and waist circumference between five groups was tested by Kruskal-Wallis test. One-way Anova test was used to compare BMI. Chi-square test was used to compare other factors among five groups.

|  |  | Sex-specific Quintiles of total lean mass | | |  |
| --- | --- | --- | --- | --- | --- |
|  |  | Q1^#^ | Q3^#^ | Q5^#^ | P* |
| N |  | 616 | 604 | 597 |  |
| Age (median [IQR]) |  | 52.00 [46.00, 56.00] | 51.00 [45.00, 56.00] | 50.00 [44.00, 55.00] | **<0.001** |
| Sex (%) | Women | 343(55.7) | 345(57.1) | 335(56.1) | 0.984 |
|  | Men | 273(44.3) | 259(42.9) | 262(43.9) |  |
| BMI (median [IQR]) |  | 20.65 [18.70, 22.80] | 22.95 [21.00, 24.70] | 25.60 [23.80, 27.70] | **<0.001** |
| Ethnicity (%) | Han nationality | 566(91.9) | 564(93.4) | 555(93.0) | 0.830 |
|  | Ethnic minority | 50(8.1) | 40(6.6) | 42(7.0) |  |
| Marital (%) | Married | 445(72.2) | 427(70.7) | 396(66.3) | 0.070 |
|  | Never married/Separated | 171(27.8) | 177(29.3) | 201(33.7) |  |
| Residence (%) | Rural | 355(57.6) | 337(55.8) | 332(55.6) | 0.569 |
|  | Urban | 261(42.4) | 267(44.2) | 265(44.4) |  |
| Education (%) | Less than high school | 327(53.1) | 299(49.5) | 285(47.7) |  |
|  | High school or equivalent | 179(29.1) | 173(28.6) | 184(30.8) | **<0.001** |
|  | College or above | 110(17.9) | 132(21.9) | 128(21.4) |  |
| Alcohol (%) | Yes | 109(17.7) | 105(17.4) | 109(18.3) | 0.591 |
| Smoke (%) | Yes | 242(39.3) | 210(34.8) | 212(35.5) | 0.992 |
| Hypertension (%) | Yes | 53(8.6) | 68(11.3) | 107(17.9) | 0.848 |
| CHD (%) | Yes | 21(3.4) | 18(3.0) | 19(3.2) | 0.619 |
| Family history of tumor (%) | Yes | 105(17.0) | 120(19.9) | 107(17.9) | 0.422 |
| Stage(%) | Advanced stage | 191(31.0) | 183(30.3) | 140(23.5) | **0.014** |
| Tumor type (%) | Lung cancer | 171(27.8) | 208(34.4) | 160(26.8) | **<0.001** |
|  | Gastrointestinal cancer | 198(32.1) | 154(25.5) | 119(19.9) |  |
|  | Breast cancer | 108(17.5) | 132(21.9) | 163(27.3) |  |
|  | Other | 139(22.6) | 110(18.2) | 155(26.0) |  |
| Surgery (%) | Yes | 86(14.0) | 99(16.4) | 121(20.3) | **0.016** |
| Chemotherapy (%) | Yes | 399(64.8) | 408(67.5) | 409(68.5) | 0.565 |
| Radiotherapy (%) | Yes | 25(4.1) | 13(2.2) | 21(3.5) | 0.411 |
| Cachexia (%) | Yes | 204(33.1) | 109(18.0) | 62(10.4) | **<0.001** |
| Eating_difficulties (%) | Yes | 266(43.2) | 200(33.1) | 141(23.6) | **<0.001** |
| Malnutrition (%) | Yes | 360(58.4) | 284(47.0) | 228(38.2) | **<0.001** |
| Blood urea nitrogen  (median [IQR]) |  | 4.92 [3.83, 6.07] | 4.92 [3.99, 6.07] | 5.03 [4.04, 6.23] | 0.213 |
| Albumin (median [IQR]) |  | 38.60 [35.30, 42.00] | 39.90 [36.80, 42.80] | 40.20 [36.90, 43.30] | **<0.001** |
| Hand grip strength  (median [IQR]) |  | 21.38 [16.78, 28.20] | 24.45 [19.58, 32.12] | 27.30 [20.50, 36.90] | **<0.001** |
| Mid-arm muscle circumference(median [IQR]) |  | 19.79 [18.11, 21.74] | 21.29 [19.42, 22.98] | 22.66 [20.84, 24.73] | **<0.001** |
| Calf circumference  (median [IQR]) |  | 32.00 [29.50, 34.00] | 34.00 [32.00, 36.00] | 37.00 [35.00, 40.00] | **<0.001** |
| Karnofsky performance status (median [IQR]) |  | 90.0[80.0,92.5] | 90.0[90.0,100.0] | 90.0[90.0,100.0] | **<0.001** |

Table S2 Clinical characteristics of participants grouped by sex-specific of total lean mass in the INSCOC cohort

^#^Men Q1, 10.3-45kg, Q3, 48.6-51.4kg, Q5 55.3-76.7kg, Women Q1, 3.7-34.2kg, Q3, 37.3-39.5kg, Q5, 42.5-68.3kg

*The difference of age, BMI, Blood urea nitrogen, Albumin, Hand grip strength, Mid-arm muscle circumference, Calf circumference, and Karnofsky performance status between five groups were tested by Kruskal-Wallis test. Chi-square test was used to compare other factors among five groups.

Results presented with bold valued were statistically significant with all p value < 0.05.

Table S3 Comparison of discrimination of all-cause mortality with different lean mass in men.

|  | C-index | IDI | | NRI | |
| --- | --- | --- | --- | --- | --- |
|  | Value | Difference | p-Value | Difference | p-Value |
| Upper limb lean mass | 0.675(0.648, 0.701) | Ref. |  | Ref. |  |
| Upper limb lean mass/weight | 0.689(0.664, 0.714) | -0.001(-0.009, 0.006) | 0.665 | -0.126(-0.289, 0.139) | 0.454 |
| Upper limb lean mass/height | 0.665(0.639, 0.691) | 0(-0.002, 0.003) | 0.889 | -0.022(-0.311, 0.179) | 0.647 |
| Upper limb lean mass/BMI | 0.690(0.665, 0.715) | -0.003(-0.009, 0.001) | 0.156 | -0.012(-0.252, 0.17) | 0.797 |
| Lower limb lean mass | 0.659(0.634, 0.684) | Ref. |  | Ref. |  |
| Lower limb lean mass/weight | 0.668(0.643, 0.693) | 0.002(-0.025, 0.028) | 0.879 | 0.004(-0.086, 0.102) | 0.927 |
| Lower limb lean mass/height | 0.656(0.630, 0.682) | -0.002(-0.012, 0.008) | 0.735 | -0.016(-0.099, 0.059) | 0.665 |
| Lower limb lean mass/BMI | 0.661(0.636, 0.686) | -0.007(-0.027, 0.012) | 0.480 | 0.002(-0.09, 0.087) | 0.117 |
| Trunk lean mass | 0.566(0.539, 0.593) | Ref. |  | Ref. |  |
| Trunk lean mass/weight | 0.499(0.471, 0.526) | -0.009(-0.017, -0.002) | **0.004** | -0.113(-0.174, -0.035) | **0.010** |
| Trunk lean mass/height | 0.530(0.503, 0.557) | -0.007(-0.013, -0.003) | **<0.001** | -0.126(-0.179, -0.058) | **0.002** |
| Trunk lean mass/BMI | 0.558(0.531, 0.585) | -0.003(-0.013, 0.005) | 0.440 | -0.031(-0.107, 0.069) | 0.601 |
| Total lean mass | 0.620(0.594, 0.647) |  |  |  |  |
| Total lean mass/weight | 0.608(0.581, 0.634) | -0.009(-0.032, 0.013) | 0.484 | -0.042(-0.131, 0.043) | 0.382 |
| Total lean mass/height | 0.600(0.573, 0.627) | -0.011(-0.021, -0.003) | **0.008** | -0.127(-0.178, -0.021) | **0.016** |
| Total lean mass/BMI | 0.624(0.598, 0.650) | -0.004(-0.021, 0.013) | 0.635 | 0(-0.091, 0.092) | 0.969 |
| Android lean mass | 0.581(0.553, 0.609) | Ref. |  | Ref. |  |
| Android lean mass/weight | 0.526(0.498, 0.553) | -0.018(-0.034, -0.007) | **<0.001** | -0.115(-0.189, -0.054) | **0.002** |
| Android lean mass/height | 0.492(0.464, 0.520) | -0.019(-0.036, -0.008) | **<0.001** | -0.124(-0.196, -0.056) | **0.002** |
| Android lean mass/BMI | 0.517(0.488, 0.545) | -0.018(-0.031, -0.008) | **<0.001** | -0.149(-0.214, -0.074) | **<0.001** |
| Gynoid lean mass | 0.723(0.700, 0.746) | Ref. |  | Ref. |  |
| Gynoid lean mass/weight | 0.678(0.653, 0.704) | -0.037(-0.067, -0.009) | **0.012** | -0.127(-0.209, -0.037) | **0.004** |
| Gynoid lean mass/height | 0.679(0.654, 0.705) | -0.031(-0.061, <0.001) | 0.052 | -0.081(-0.165, 0.008) | 0.078 |
| Gynoid lean mass/BMI | 0.701(0.677, 0.725) | -0.022(-0.037, -0.007) | **0.002** | -0.156(-0.237, -0.055) | **0.004** |

Table S4 Comparison of discrimination of all-cause mortality with different lean mass in women.

|  | C-index | IDI | | NRI | |
| --- | --- | --- | --- | --- | --- |
|  | Value | Difference | p-Value | Difference | p-Value |
| Upper limb lean mass | 0.609(0.578, 0.640) | Ref. |  | Ref. |  |
| Upper limb lean mass/weight | 0.591(0.560, 0.623) | -0.007(-0.019, 0.006) | 0.310 | -0.038(-0.142, 0.059) | 0.416 |
| Upper limb lean mass/height | 0.592(0.561, 0.623) | -0.006(-0.010, -0.003) | **<0.001** | -0.214(-0.272, -0.146) | **<0.001** |
| Upper limb lean mass/BMI | 0.568(0.537, 0.598) | -0.012(-0.02, -0.006) | **<0.001** | -0.210(-0.268, -0.129) | **<0.001** |
| Lower limb lean mass | 0.628(0.597, 0.658) |  |  |  |  |
| Lower limb lean mass/weight | 0.638(0.608, 0.669) | 0.006(-0.008, 0.025) | 0.452 | 0.073(-0.025, 0.180) | 0.174 |
| Lower limb lean mass/height | 0.615(0.584, 0.646) | -0.005(-0.008, -0.002) | **<0.001** | -0.206(0.266, -0.096) | **<0.001** |
| Lower limb lean mass/BMI | 0.594(0.564, 0.625) | -0.012(-0.020, -0.006) | **<0.001** | -0.209(-0.283, -0.148) | **<0.001** |
| Trunk lean mass | 0.566(0.534, 0.598) | Ref. |  | Ref. |  |
| Trunk lean mass/weight | 0.514(0.484, 0.545) | -0.006(-0.014, 0) | **0.032** | -0.093(-0.153, 0.008) | 0.072 |
| Trunk lean mass/height | 0.542(0.510, 0.573) | -0.004(-0.008, -0.001) | **0.002** | -0.150(-0.214, -0.058) | **<0.001** |
| Trunk lean mass/BMI | 0.510(0.480, 0.541) | -0.006(-0.012, 0) | **0.040** | -0.108(-0.178, -0.005) | **0.042** |
| Total lean mass | 0.599(0.567, 0.630) |  |  | Ref. |  |
| Total lean mass/weight | 0.574(0.543, 0.605) | -0.008(-0.023, 0.004) | 0.170 | -0.035(-0.148, 0.048) | 0.330 |
| Total lean mass/height | 0.579(0.548, 0.610) | -0.006(-0.01, -0.003) | **<0.001** | -0.197(-0.264, -0.127) | **<0.001** |
| Total lean mass/BMI | 0.548(0.518, 0.579) | -0.012(-0.02, -0.005) | **<0.001** | -0.172(-0.250, -0.102) | **<0.001** |
| Android lean mass | 0.566(0.533, 0.599) | Ref. |  | Ref. |  |
| Android lean mass/weight | 0.516(0.485, 0.547) | -0.009(-0.018, -0.003) | **<0.001** | -0.158(-0.213, -0.042) | **0.004** |
| Android lean mass/height | 0.538(0.509, 0.568) | -0.008(-0.019, -0.002) | **<0.001** | -0.113(-0.196, 0.006) | 0.060 |
| Android lean mass/BMI | 0.506(0.475, 0.538) | -0.009(-0.02, -0.002) | **0.002** | -0.146(-0.220, -0.037) | **0.002** |
| Gynoid lean mass | 0.664(0.635, 0.693) | Ref. |  | Ref. |  |
| Gynoid lean mass/weight | 0.632(0.602, 0.662) | -0.007(-0.024, 0.009) | 0.404 | -0.061(-0.157, 0.038) | 0.272 |
| Gynoid lean mass/height | 0.642(0.612, 0.673) | -0.003(-0.019, 0.014) | 0.767 | -0.024(-0.137, 0.07) | 0.555 |
| Gynoid lean mass/BMI | 0.611(0.581, 0.641) | -0.014(-0.031, 0) | 0.054 | -0.102(-0.19, 0.018) | 0.116 |

Table S5 Hazards ratio (95% CI) for cause-specific mortality of lean mass

|  | Died of cardiovascular disease |  | Died of Cancer |  | Died of other diseases |  |
| --- | --- | --- | --- | --- | --- | --- |
|  | HR (95%CI) | P | HR (95%CI) | P | HR (95%CI) | P |
| Events/Total | 267/5052 |  | 206/5052 |  | 353/5052 |  |
| Mortality rate per 1000-year | 3.78 |  | 2.92 |  | 5 |  |
| Upper limb lean mass |  |  |  |  |  |  |
| Q1 | Ref. |  | Ref. |  | Ref. |  |
| Q2 | 0.55(0.37, 0.81) | **0.003** | **0.56(0.36, 0.85)** | **0.006** | 0.62(0.38, 1.01) | 0.055 |
| Q3 | 0.28(0.16, 0.47) | **<0.001** | 0.63(0.38, 1.03) | 0.065 | 0.42(0.26, 0.66) | **<0.001** |
| Q4 | 0.22(0.12, 0.39) | **<0.001** | **0.50(0.32, 0.79)** | **0.003** | 0.49(0.28, 0.86) | **0.013** |
| Q5 | 0.27(0.15, 0.49) | **<0.001** | **0.45(0.31, 0.65)** | **<0.001** | 0.34(0.16, 0.71) | **0.004** |
| P for trend |  | **<0.001** |  | **<0.001** |  | **0.001** |
| As continuous (per SD) | 0.38(0.29, 0.51) | **<0.001** | **0.47(0.37, 0.60)** | **<0.001** | 0.46(0.28, 0.75) | **0.002** |
| Lower limb lean mass |  |  |  |  |  |  |
| Q1 | Ref. |  | Ref. |  | Ref. |  |
| Q2 | 0.48(0.31, 0.75) | **0.001** | 0.73(0.45, 1.18) | 0.202 | 0.52(0.37, 0.73) | **<0.001** |
| Q3 | 0.36(0.21, 0.64) | **<0.001** | 0.53(0.29, 0.97) | 0.038 | 0.43(0.28, 0.67) | **<0.001** |
| Q4 | 0.28(0.16, 0.49) | **<0.001** | 0.63(0.35, 1.14) | 0.126 | 0.42(0.24, 0.74) | **0.003** |
| Q5 | 0.35(0.18, 0.66) | **0.001** | 0.45(0.19, 1.06) | 0.069 | 0.22(0.13, 0.39) | **<0.001** |
| P for trend |  | **<0.001** |  | 0.059 |  | **<0.001** |
| As continuous (per SD) | 0.60(0.45, 0.80) | **<0.001** | 0.63(0.44, 0.90) | 0.066 | 0.51(0.34, 0.75) | **0.001** |
| U-L ratio |  |  |  |  |  |  |
| Q1 | Ref. |  | Ref. |  | Ref. |  |
| Q2 | 0.42(0.24, 0.72) | **0.002** | 0.58(0.33, 1.01) | 0.053 | 0.66(0.39, 1.12) | 0.126 |
| Q3 | 0.62(0.37, 1.02) | 0.060 | 0.92(0.48, 1.75) | 0.794 | 0.82(0.52, 1.29) | 0.386 |
| Q4 | 0.45(0.28, 0.73) | **0.001** | 0.57(0.31, 1.03) | 0.061 | 0.66(0.39, 1.12) | 0.126 |
| Q5 | 0.33(0.21, 0.53) | **<0.001** | 0.55(0.29, 1.06) | 0.075 | 0.78(0.48, 1.27) | 0.320 |
| P for trend |  | **<0.001** |  | 0.125 |  | 0.381 |
| As continuous (per SD) | 0.60(0.47, 0.77) | **<0.001** | 0.88(0.69, 1.13) | 0.318 | 0.90(0.77, 1.04) | 0.152 |
| Trunk lean mass |  |  |  |  |  |  |
| Q1 | Ref. |  | Ref. |  | Ref. |  |
| Q2 | 0.58(0.36, 0.95) | **0.029** | 0.52(0.29, 0.92) | **0.025** | 0.78(0.46, 1.32) | 0.347 |
| Q3 | 0.44(0.29, 0.65) | **<0.001** | 0.61(0.36, 1.04) | 0.071 | 0.91(0.52, 1.59) | 0.737 |
| Q4 | 0.48(0.31, 0.74) | **0.001** | 0.73(0.41, 1.27) | 0.262 | 0.46(0.26, 0.82) | **0.009** |
| Q5 | 0.39(0.21, 0.71) | **0.002** | 0.59(0.27, 1.28) | 0.181 | 0.52(0.26, 1.03) | 0.062 |
| P for trend |  | **0.001** |  | 0.400 |  | **0.013** |
| As continuous (per SD) | 0.74(0.55, 0.99) | **0.040** | 0.76(0.58, 1.00) | **0.046** | 0.83(0.72, 0.96) | **0.012** |
| Total lean mass |  |  |  |  |  |  |
| Q1 | Ref. |  | Ref. |  | Ref. |  |
| Q2 | 0.51(0.36, 0.73) | **<0.001** | 0.59(0.37, 0.95) | **0.031** | 0.60(0.37, 1) | **0.048** |
| Q3 | 0.36(0.23, 0.57) | **<0.001** | 0.53(0.30, 0.92) | **0.025** | 0.58(0.36, 0.93) | **0.023** |
| Q4 | 0.33(0.19, 0.57) | **<0.001** | 0.70(0.40, 1.20) | 0.194 | 0.47(0.26, 0.85) | **0.012** |
| Q5 | 0.30(0.15, 0.61) | **0.001** | 0.49(0.24, 0.99) | **0.047** | 0.30(0.14, 0.64) | **0.002** |
| P for trend |  | **<0.001** |  | 0.160 |  | **0.002** |
| As continuous (per SD) | 0.58(0.43, 0.78) | **<0.001** | 0.62(0.46, 0.84) | **0.002** | 0.54(0.35, 0.84) | **0.022** |
| Android lean mass |  |  |  |  |  |  |
| Q1 | Ref. |  | Ref. |  | Ref. |  |
| Q2 | 0.68(0.29, 1.60) | 0.374 | 0.84(0.48, 1.47) | 0.534 | 0.68(0.37, 1.28) | 0.233 |
| Q3 | 1.06(0.57, 1.99) | 0.846 | 1.02(0.59, 1.74) | 0.954 | 0.74(0.42, 1.32) | 0.308 |
| Q4 | 0.89(0.54, 1.46) | 0.642 | 0.73(0.37, 1.45) | 0.370 | 0.71(0.35, 1.45) | 0.351 |
| Q5 | 1.41(0.75, 2.65) | 0.283 | 1.22(0.61, 2.42) | 0.578 | 0.62(0.29, 1.36) | 0.235 |
| P for trend |  | 0.051 |  | 0.716 |  | 0.328 |
| As continuous (per SD) | 1.85(1.56, 2.20) | **<0.001** | 1.01(0.82, 1.24) | 0.904 | 0.91(0.72, 1.15) | 0.430 |
| Gynoid lean mass |  |  |  |  |  |  |
| Q1 | Ref. |  | Ref. |  | Ref. |  |
| Q2 | 0.32(0.23, 0.44) | **<0.001** | 0.60(0.32, 1.12) | 0.107 | 0.57(0.41, 0.78) | **0.001** |
| Q3 | 0.33(0.23, 0.47) | **<0.001** | 0.56(0.36, 0.87) | **0.010** | 0.42(0.25, 0.70) | **0.001** |
| Q4 | 0.28(0.18, 0.44) | **<0.001** | 0.61(0.38, 0.97) | **0.036** | 0.37(0.17, 0.80) | **0.011** |
| Q5 | 0.17(0.10, 0.28) | **<0.001** | 0.54(0.28, 1.04) | 0.064 | 0.22(0.09, 0.52) | **0.001** |
| P for trend |  | **<0.001** |  | **0.050** |  | **0.001** |
| As continuous (per SD) | 0.44(0.35, 0.56) | **<0.001** | 0.57(0.43, 0.76) | **<0.001** | 0.49(0.30, 0.80) | **0.004** |
| A-G ratio |  |  |  |  |  |  |
| Q1 | Ref. |  | Ref. |  | Ref. |  |
| Q2 | 1.48(0.62, 3.54) | 0.378 | 0.71(0.32, 1.55) | 0.385 | 1.23(0.65, 2.33) | 0.519 |
| Q3 | 2.10(0.86, 5.15) | 0.105 | 0.67(0.24, 1.85) | 0.442 | 1.11(0.48, 2.58) | 0.809 |
| Q4 | 2.45(1.03, 5.86) | **0.044** | 1.70(0.63, 4.61) | 0.297 | 1.53(0.71, 3.31) | 0.277 |
| Q5 | 6.41(2.56, 16.08) | **<0.001** | 1.77(0.74, 4.25) | 0.202 | 2.95(1.45, 5.99) | **0.003** |
| P for trend |  | **<0.001** |  | **0.001** |  | **<0.001** |
| As continuous (per SD) | 1.66(1.44, 1.91) | **<0.001** | 1.45(1.22, 1.72) | **<0.001** | 1.46(1.27, 1.67) | **<0.001** |

Table S6 Additional analyses

|  | Additional analysis 1 | | Additional analysis 2 | | Additional analysis 3 | | Additional analysis 4 | |
| --- | --- | --- | --- | --- | --- | --- | --- | --- |
|  | HR (95%CI) | P | HR (95%CI) | P | HR (95%CI) | P | HR (95%CI) | P |
| Events/Total | 777/5003 |  |  |  |  |  |  |  |
| Mortality rate per 1000 | 11.09 |  |  |  |  |  |  |  |
| Upper limb lean mass |  |  |  |  |  |  |  |  |
| Q1 | Ref. |  | Ref. |  | Ref. |  | Ref. |  |
| Q2 | 0.61(0.46, 0.81) | **0.001** | 0.51(0.3, 0.87) | **0.014** | 0.51(0.35, 0.73) | **<0.001** | 0.56(0.42, 0.75) | **<0.001** |
| Q3 | 0.48(0.36, 0.64) | **<0.001** | 0.31(0.18, 0.54) | **<0.001** | 0.38(0.25, 0.57) | **<0.001** | 0.42(0.31, 0.57) | **<0.001** |
| Q4 | 0.41(0.30, 0.56) | **<0.001** | 0.26(0.14, 0.51) | **<0.001** | 0.35(0.22, 0.55) | **<0.001** | 0.37(0.26, 0.52) | **<0.001** |
| Q5 | 0.37(0.27, 0.51) | **<0.001** | 0.18(0.11, 0.29) | **<0.001** | 0.35(0.22, 0.55) | **<0.001** | 0.33(0.24, 0.47) | **<0.001** |
| P for trend | 0.77(0.72, 0.83) | **<0.001** |  | **<0.001** |  | **<0.001** |  | **<0.001** |
| As continuous (per SD) | 0.78(0.70, 0.87) | **<0.001** | 0.23(0.16, 0.33) | **<0.001** | 0.4(0.28, 0.55) | **<0.001** | 0.42(0.33, 0.53) | **<0.001** |
| Lower limb lean mass |  |  |  |  |  |  |  |  |
| Q1 | Ref. |  | Ref. |  | Ref. |  | Ref. |  |
| Q2 | 0.61(0.47, 0.79) | **<0.001** | 0.28(0.19, 0.41) | **<0.001** | 0.44(0.33, 0.6) | **<0.001** | 0.56(0.44, 0.71) | **<0.001** |
| Q3 | 0.45(0.34, 0.61) | **<0.001** | 0.35(0.21, 0.58) | **<0.001** | 0.41(0.29, 0.59) | **<0.001** | 0.47(0.35, 0.63) | **<0.001** |
| Q4 | 0.43(0.31, 0.60) | **<0.001** | 0.19(0.11, 0.33) | **<0.001** | 0.38(0.25, 0.59) | **<0.001** | 0.43(0.31, 0.6) | **<0.001** |
| Q5 | 0.33(0.25, 0.44) | **<0.001** | 0.18(0.09, 0.38) | **<0.001** | 0.3(0.18, 0.48) | **<0.001** | 0.33(0.24, 0.45) | **<0.001** |
| P for trend |  | **<0.001** |  | **<0.001** |  | **<0.001** |  | **<0.001** |
| As continuous (per SD) | 0.57(0.48, 0.68) | **<0.001** | 0.42(0.31, 0.56) | **<0.001** | 0.52(0.41, 0.66) | **<0.001** | 0.57(0.48, 0.67) | **<0.001** |
| U-L ratio |  |  |  |  |  |  |  |  |
| Q1 | Ref. |  | Ref. |  | Ref. |  | Ref. |  |
| Q2 | 0.6(0.39, 0.92) | **0.019** | 0.6(0.31, 1.19) | 0.146 | 0.65(0.42, 1.02) | 0.059 | 0.56(0.37, 0.85) | **0.007** |
| Q3 | 0.84(0.59, 1.20) | 0.343 | 0.6(0.34, 1.08) | 0.087 | 0.83(0.55, 1.26) | 0.386 | 0.76(0.51, 1.14) | 0.179 |
| Q4 | 0.62(0.47, 0.80) | **<0.001** | 0.49(0.28, 0.86) | **0.014** | 0.63(0.42, 0.92) | **0.018** | 0.55(0.41, 0.73) | **<0.001** |
| Q5 | 0.61(0.44, 0.86) | **0.005** | 0.39(0.17, 0.91) | **0.029** | 0.6(0.38, 0.95) | **0.030** | 0.53(0.37, 0.74) | **<0.001** |
| P for trend |  | **0.005** |  | **0.009** |  | **0.035** |  | **<0.001** |
| As continuous (per SD) | 0.80(0.69, 0.91) | **0.001** | 0.55(0.4, 0.76) | **<0.001** | 0.74(0.59, 0.93) | **0.009** | 0.72(0.62, 0.84) | **<0.001** |
| Trunk lean mass |  |  |  |  |  |  |  |  |
| Q1 | Ref. |  | Ref. |  | Ref. |  | Ref. |  |
| Q2 | 0.63(0.47, 0.83) | **0.001** | 0.97(0.52, 1.79) | 0.921 | 0.69(0.47, 1.01) | 0.059 | 0.64(0.47, 0.87) | **0.004** |
| Q3 | 0.68(0.47, 0.98) | **0.039** | 0.57(0.31, 1.05) | 0.072 | 0.52(0.33, 0.82) | **0.005** | 0.64(0.43, 0.94) | **0.025** |
| Q4 | 0.59(0.44, 0.8) | **0.001** | **0.48(0.29, 0.78)** | **0.003** | 0.51(0.34, 0.75) | **0.001** | 0.56(0.4, 0.79) | **0.001** |
| Q5 | 0.49(0.32, 0.76) | **0.001** | **0.39(0.18, 0.84)** | **0.016** | 0.49(0.27, 0.9) | **0.021** | 0.50(0.32, 0.79) | **0.003** |
| P for trend |  | **0.001** |  | **<0.001** |  | **0.002** |  | **0.002** |
| As continuous (per SD) | 0.71(0.59, 0.85) | **<0.001** | **0.54(0.4, 0.74)** | **<0.001** | 0.7(0.53, 0.94) | **0.017** | 0.72(0.59, 0.87) | **0.001** |
| Total lean mass |  |  |  |  |  |  |  |  |
| Q1 | Ref. |  | **Ref.** |  | Ref. |  | Ref. |  |
| Q2 | 0.61(0.45, 0.83) | **0.001** | **0.5(0.31, 0.81)** | **0.005** | 0.46(0.32, 0.66) | **<0.001** | 0.55(0.41, 0.74) | **<0.001** |
| Q3 | 0.51(0.37, 0.71) | **<0.001** | **0.41(0.26, 0.67)** | **<0.001** | 0.42(0.30, 0.60) | **<0.001** | 0.49(0.35, 0.68) | **<0.001** |
| Q4 | 0.51(0.37, 0.71) | **<0.001** | **0.26(0.15, 0.45)** | **<0.001** | 0.39(0.26, 0.58) | **<0.001** | 0.49(0.34, 0.71) | **<0.001** |
| Q5 | 0.36(0.24, 0.54) | **<0.001** | **0.19(0.1, 0.37)** | **<0.001** | 0.36(0.21, 0.61) | **<0.001** | 0.35(0.24, 0.53) | **<0.001** |
| P for trend |  | **<0.001** |  | **<0.001** |  | **<0.001** |  | **<0.001** |
| As continuous (per SD) | 0.57(0.47, 0.69) | **<0.001** | **0.39(0.29, 0.51)** | **<0.001** | 0.53(0.40, 0.71) | **<0.001** | 0.56(0.46, 0.69) | **<0.001** |
| Android lean mass |  |  |  |  |  |  |  |  |
| Q1 | Ref. |  | Ref. |  | Ref. |  | Ref. |  |
| Q2 | 0.65(0.45, 0.95) | **0.024** | 0.84(0.45, 1.59) | 0.596 | 0.66(0.41, 1.07) | 0.090 | 0.65(0.43, 0.98) | **0.042** |
| Q3 | 0.76(0.58, 0.98) | **0.038** | 0.69(0.38, 1.28) | 0.242 | 0.72(0.50, 1.05) | 0.087 | 0.76(0.57, 1.02) | 0.067 |
| Q4 | 0.59(0.40, 0.86) | **0.007** | 0.61(0.26, 1.42) | 0.248 | 0.51(0.31, 0.83) | **0.007** | 0.60(0.39, 0.91) | **0.017** |
| Q5 | 0.69(0.44, 1.08) | 0.106 | 0.78(0.34, 1.79) | 0.552 | 0.79(0.48, 1.31) | 0.358 | 0.74(0.47, 1.17) | 0.2 |
| P for trend |  | 0.067 |  | 0.442 |  | 0.229 |  | 0.191 |
| As continuous (per SD) | 0.91(0.79, 1.06) | 0.224 | 0.82(0.53, 1.26) | 0.375 | 0.93(0.72, 1.20) | 0.578 | 0.97(0.80, 1.18) | 0.765 |
| Gynoid lean mass |  |  |  |  |  |  |  |  |
| Q1 | Ref. |  | Ref. |  | Ref. |  | Ref. |  |
| Q2 | 0.56(0.43, 0.73) | <0.001 | 0.29(0.19, 0.46) | <0.001 | 0.37(0.25, 0.57) | **<0.001** | 0.48(0.37, 0.63) | **<0.001** |
| Q3 | 0.45(0.34, 0.58) | <0.001 | 0.27(0.16, 0.44) | <0.001 | 0.35(0.24, 0.51) | **<0.001** | 0.41(0.31, 0.54) | **<0.001** |
| Q4 | 0.39(0.28, 0.57) | <0.001 | 0.15(0.08, 0.31) | <0.001 | 0.27(0.18, 0.40) | **<0.001** | 0.38(0.25, 0.56) | **<0.001** |
| Q5 | 0.27(0.18, 0.42) | <0.001 | 0.11(0.05, 0.25) | <0.001 | 0.20(0.11, 0.34) | **<0.001** | 0.26(0.17, 0.39) | **<0.001** |
| P for trend |  | <0.001 |  | <0.001 |  | **<0.001** |  | **<0.001** |
| As continuous (per SD) | 0.48(0.39, 0.59) | <0.001 | 0.26(0.18, 0.38) | <0.001 | 0.43(0.32, 0.57) | **<0.001** | 0.48(0.39, 0.59) | **<0.001** |
| A-G ratio |  |  |  |  |  |  |  |  |
| Q1 | Ref. |  | Ref. |  | Ref. |  | Ref. |  |
| Q2 | 1(0.67, 1.50) | 0.988 | 1.43(0.54, 3.76) | 0.468 | 1.49(0.87, 2.53) | 0.144 | 0.93(0.58, 1.49) | 0.762 |
| Q3 | 1.07(0.63, 1.83) | 0.792 | 3.4(1.15, 10.04) | 0.026 | 1.30(0.67, 2.51) | 0.432 | 1.05(0.64, 1.74) | 0.835 |
| Q4 | 1.80(1.07, 3.03) | 0.027 | 4.41(1.64, 11.84) | 0.003 | 2.12(1.14, 3.93) | **0.017** | 1.66(1.01, 2.74) | **0.047** |
| Q5 | 2.92(1.75, 4.89) | <0.001 | 13.08(4.72, 36.29) | <0.001 | 4.41(2.60, 7.49) | **<0.001** | 3.04(1.95, 4.74) | **<0.001** |
| P for trend |  | <0.001 |  | <0.001 |  | **<0.001** |  | **<0.001** |
| As continuous (per SD) | 1.5(1.34, 1.68) | <0.001 | 1.92(1.58, 2.32) | <0.001 | 1.64(1.43, 1.88) | **<0.001** | 1.55(1.41, 1.72) | **<0.001** |

Additional analysis 1 Excluding participants who died in 18 months

Additional analysis 2 The relationship between muscle distribution and 5-year mortality of participants

Additional analysis 3 The relationship between muscle distribution and 10-year mortality of participants

Additional analysis 4 The relationship between muscle distribution and 15-year mortality of participants

Adjusted for age, sex, race/ethnicity, education level, marital status, family income-poverty ratio level, hypertension, CHD, diabetes, cancer, smoke, [covered by health insurance](https://wwwn.cdc.gov/Nchs/Nhanes/2003-2004/HIQ_C.htm#HID010), alcohol, BMI, Waist circumference, [muscle strengthening activities](https://wwwn.cdc.gov/Nchs/Nhanes/2003-2004/PAQ_C.htm#PAD440), HEI 2015.

All quintiles (Q1-5) were represented by sex-specific quintiles.

Table S7 The relationship between total lean mass and prognosis of patients with cancer in the INSCOC cohort

|  | Model 1 |  | Model 2 |  |
| --- | --- | --- | --- | --- |
|  | HR (95%CI) | P | HR (95%CI) | P |
| Total lean mass (per SD) | 0.911(0.847, 0.979) | **0.011** | 0.915(0.841, 0.995) | **0.037** |
| Q1 | Ref. |  | Ref. |  |
| Q2 | 0.701(0.578, 0.849) | **0.000** | 0.782(0.641, 0.954) | **0.016** |
| Q3 | 0.719(0.593, 0.871) | **0.001** | 0.800(0.651, 0.983) | **0.034** |
| Q4 | 0.621(0.510, 0.756) | **0.000** | 0.759(0.594, 0.969) | **0.027** |
| Q5 | 0.565(0.461, 0.692) | **0.000** | 0.675(0.539, 0.847) | **0.001** |
| p for trend |  | **<0.001** |  | **0.012** |
| 1-year |  |  |  |  |
| Total lean mass (per SD) | 0.760(0.686, 0.843) | **<0.001** | 0.802(0.712, 0.905) | **<0.001** |
| Q1 | Ref. |  | Ref. |  |
| Q2 | 0.628(0.478, 0.826) | **0.001** | 0.799(0.656, 0.974) | **0.026** |
| Q3 | 0.544(0.408, 0.725) | **0.000** | 0.805(0.656, 0.987) | **0.037** |
| Q4 | 0.561(0.421, 0.748) | **0.000** | 0.778(0.612, 0.989) | **0.040** |
| Q5 | 0.461(0.340, 0.625) | **0.000** | 0.661(0.528, 0.829) | **0.000** |
| p for trend |  | **<0.001** |  | **0.012** |
| 3-year |  |  |  |  |
| Total lean mass (per SD) | 0.865(0.801, 0.933) | **<0.001** | 0.879(0.806, 0.961) | **0.004** |
| Q1 | Ref. |  | Ref. |  |
| Q2 | 0.728(0.597, 0.888) | **0.002** | 0.831(0.677, 1.020) | 0.077 |
| Q3 | 0.698(0.570, 0.854) | **0.000** | 0.797(0.642, 0.989) | **0.040** |
| Q4 | 0.6(0.487, 0.739) | **0.000** | 0.766(0.591, 0.993) | **0.044** |
| Q5 | 0.507(0.407, 0.632) | **0.000** | 0.635(0.498, 0.811) | **0.000** |
| p for trend |  | **<0.001** |  | **0.006** |
| 5-year |  |  |  |  |
| Total lean mass (per SD) | 0.901(0.838, 0.970) | **0.006** | 0.907(0.834, 0.987) | **0.024** |
| Q1 | Ref. |  | Ref. |  |
| Q2 | 0.711(0.586, 0.862) | **0.001** | 0.798(0.653, 0.974) | **0.027** |
| Q3 | 0.717(0.591, 0.870) | **0.001** | 0.800(0.650, 0.984) | **0.035** |
| Q4 | 0.627(0.514, 0.764) | **0.000** | 0.772(0.603, 0.989) | **0.041** |
| Q5 | 0.544(0.442, 0.668) | **0.000** | 0.658(0.523, 0.828) | **0.000** |
| p for trend |  | **0.001** |  | **0.011** |

Results presented with bold valued were statistically significant with all p value < 0.05.

Figure S1 Flow chart of research design (NHANES and INSCOC cohort)


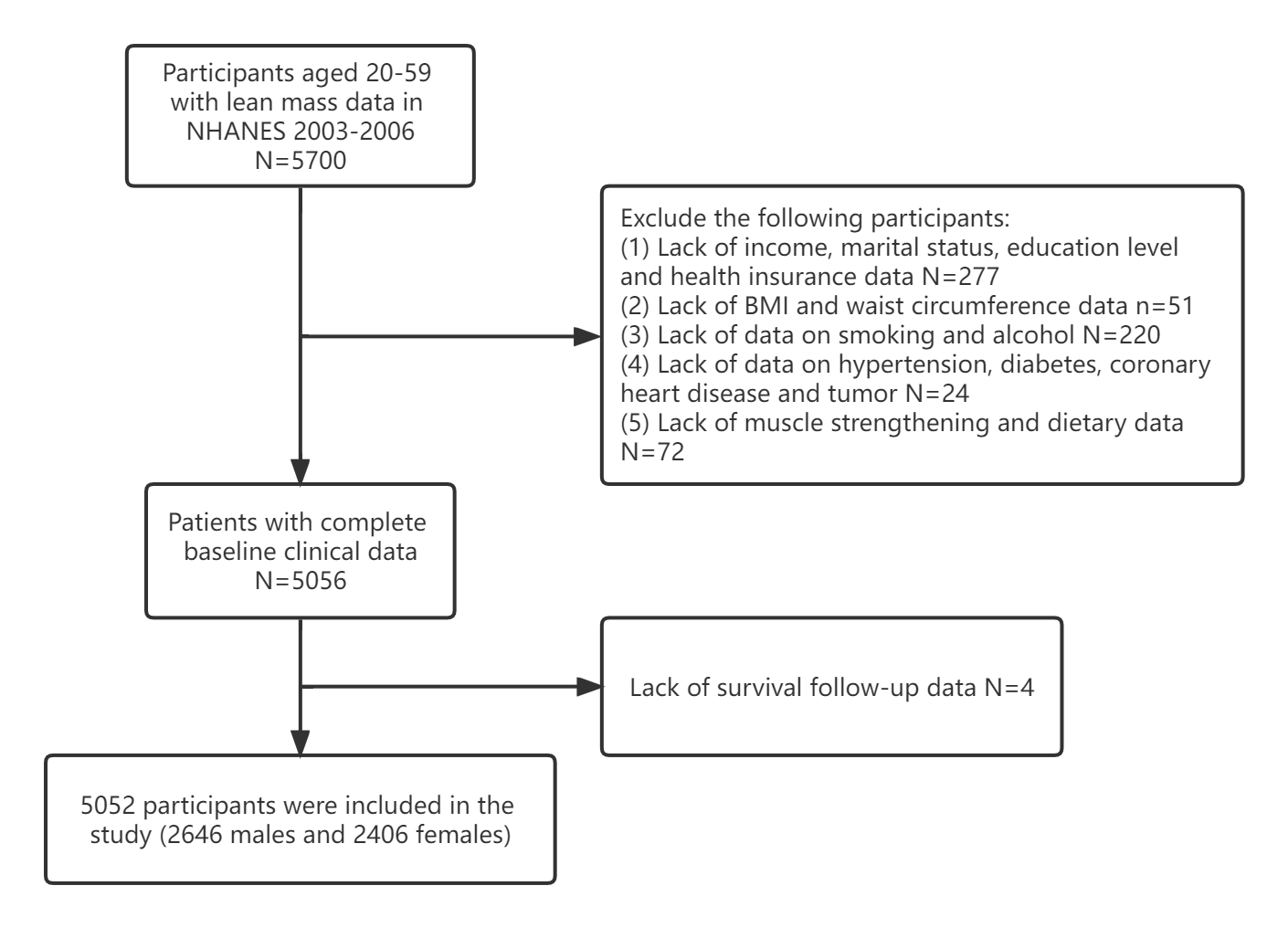


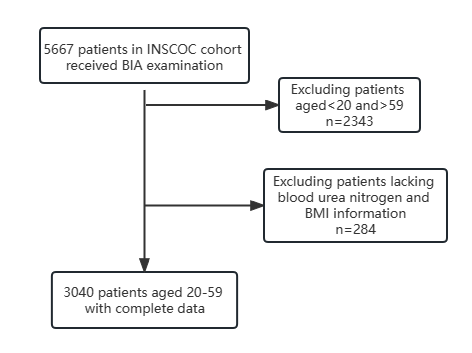


Figure S2 Kaplan-Meier Curves of sex-specific quintiles of lean mass

A Total participants


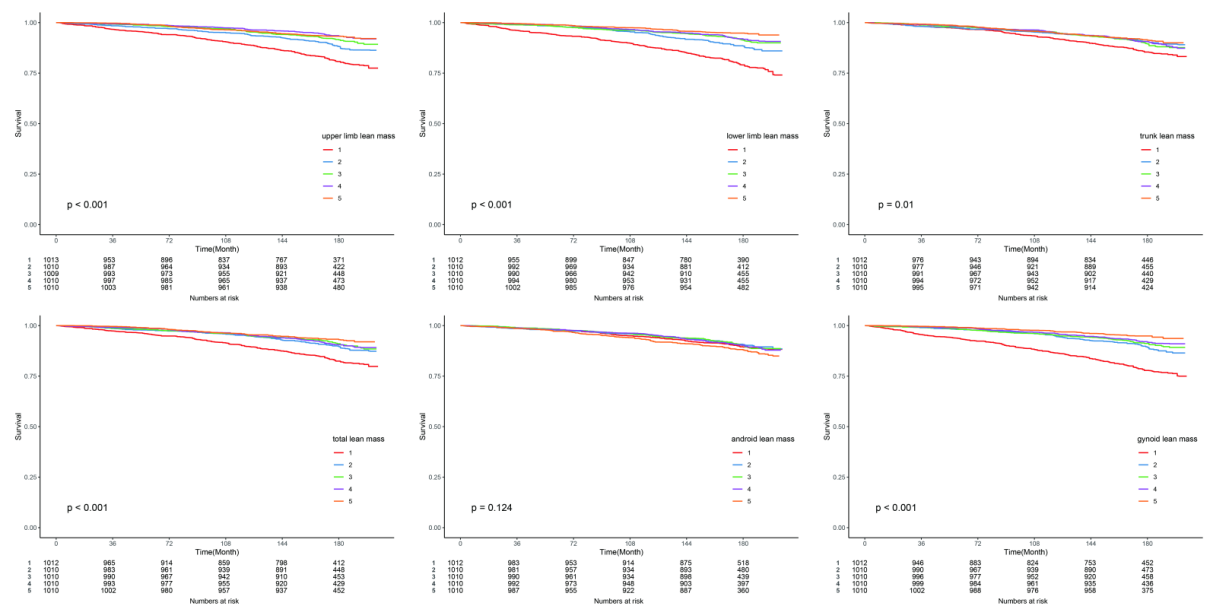


B Men


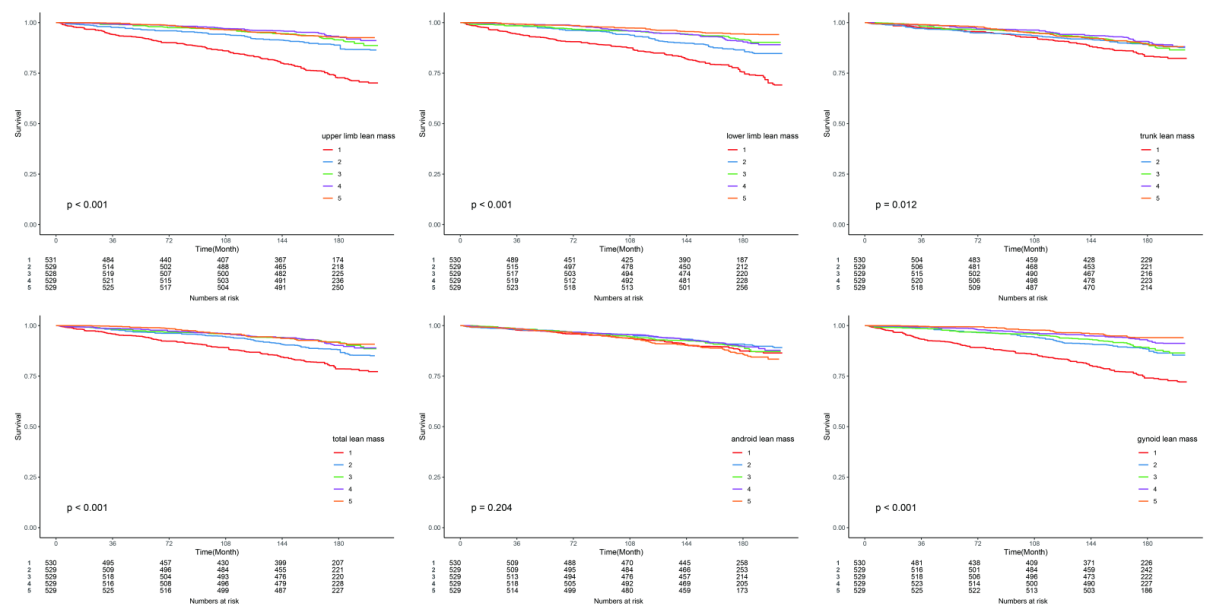


C Women


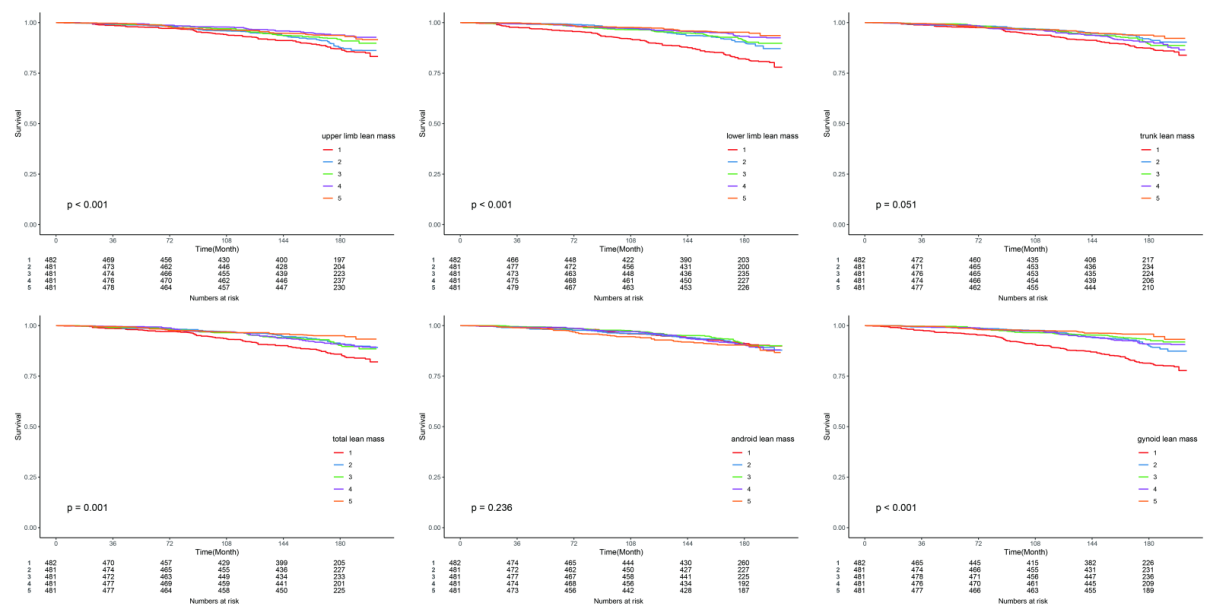


Figure S3 The relationship between lean mass and all-cause mortality in different sexes


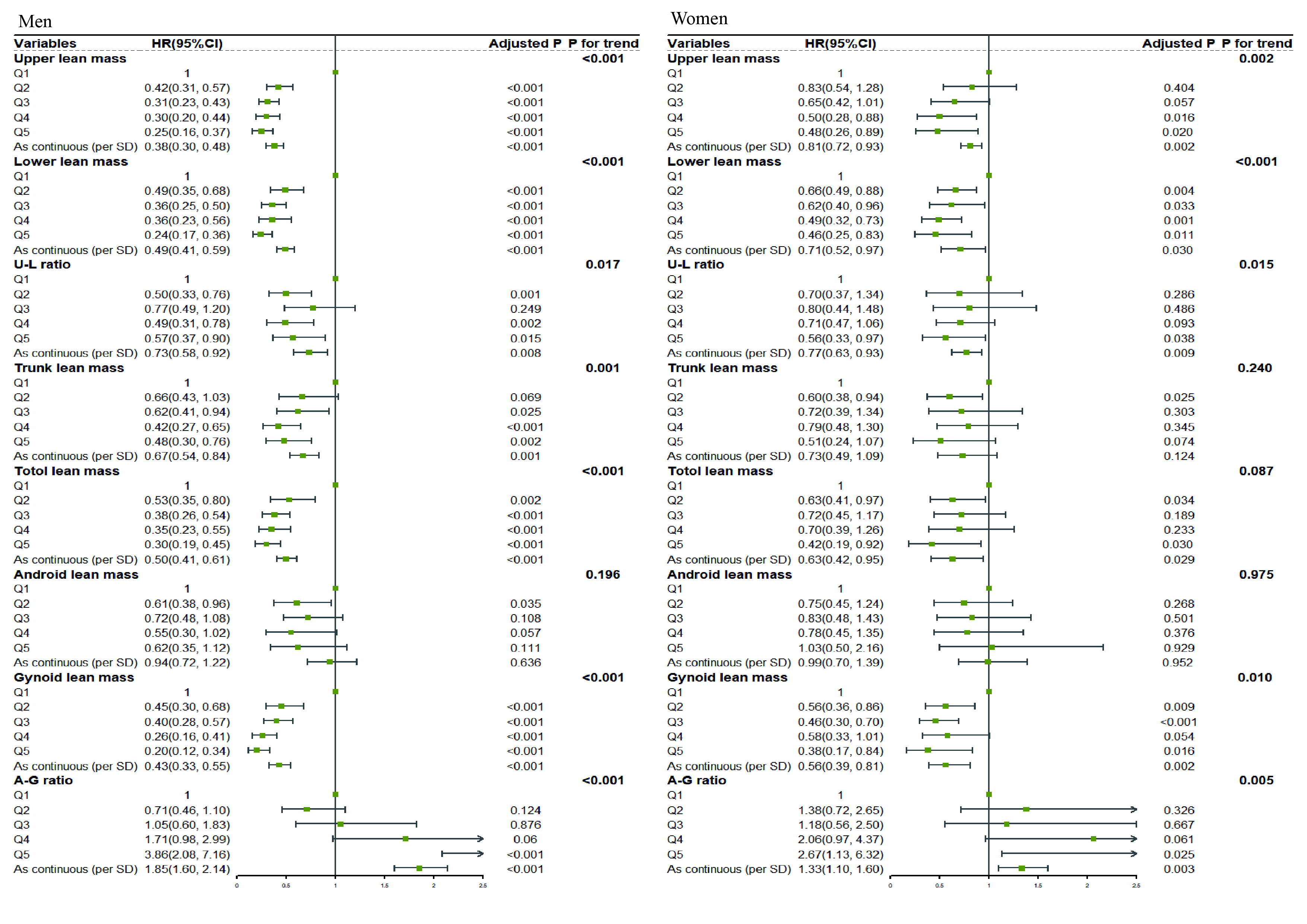


Figure S4 The relationship between lean mass and all-cause mortality in young and middle-aged participants


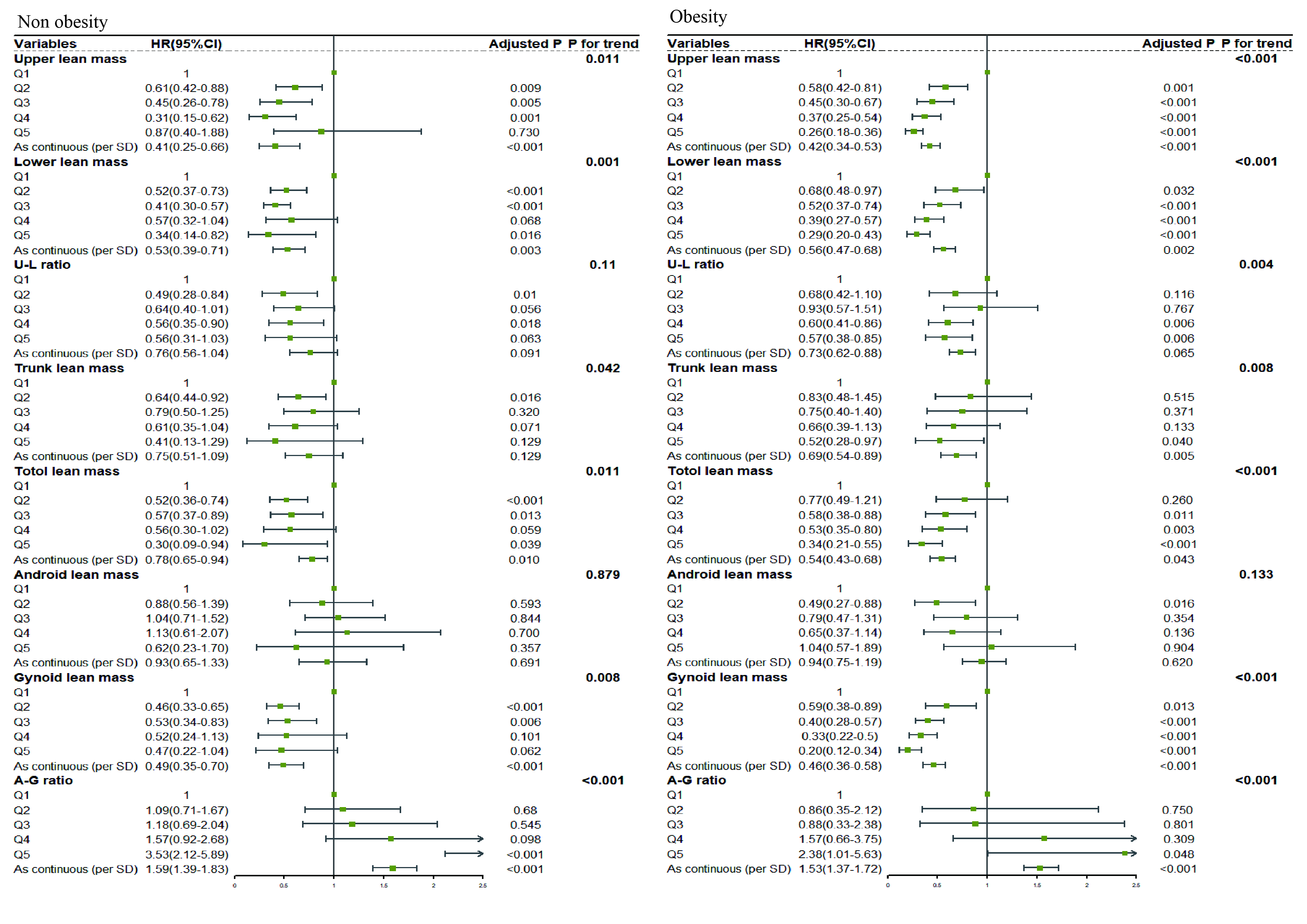

Supplement: Supplementary file 1 — Additional file 1: TableS1. Clinical characteristics of participants grouped by sex-specific ofdifferent regional lean mass. Table S2. Clinical characteristics of participants grouped bysex-specific of total lean mass in the INSCOC cohort. Table S3.Comparison of discrimination of all-cause mortality with different lean mass inmen. Table S4. Comparison of discrimination of all-cause mortality withdifferent lean mass in women. Table S5. Hazards ratio (95% CI) forcause-specific mortality of lean mass. Table S6. Additional analyses. Table S7. The relationshipbetween total lean mass and prognosis of patients with cancer in the INSCOCcohort. FigureS1. Flow chart of research design (NHANES and INSCOCcohort). Figure S2. Kaplan-Meier Curves of sex-specific quintiles of leanmass. Figure S3. The relationship betweenlean mass and all-cause mortality in different sexes. Figure S4 Therelationship between lean mass and all-cause mortality in young and middle-agedparticipants. [file 12967_2023_4008_MOESM1_ESM.docx]
